# AIM2 drives inflammatory cell death and monkeypox pathogenesis

**DOI:** 10.1038/s41423-025-01367-7

**Published:** 2025-11-12

**Authors:** Jueun Oh, Yun-Ho Hwang, Jihye Lee, Cheong Seok, SuHyeon Oh, Hye Yoon Kim, Nabukenya Mariam, Jaeyoung Ahn, GyeongJu Yu, Jaewoo Park, Hayeon Kim, Suhyun Kim, Seyun Shin, Min-Chul Jung, Jinwoo Gil, Joo Sang Lee, Young Ki Choi, Dokeun Kim, Daesik Kim, You-Jin Kim, SangJoon Lee

**Affiliations:** 1 Department of Biological Science, Ulsan National Institute of Science and Technology (UNIST), Ulsan, Republic of Korea; 2 Division of Infectious Disease Vaccine Research, Center for Vaccine Research, National Institute of Infectious Diseases, National Institute of Health, CheongJu, Chungbuk Republic of Korea; 3 Department of Precision Medicine, School of Medicine, Sungkyunkwan University, Suwon, Republic of Korea; 4 Center for Study of Emerging and Re-emerging Viruses, Korea Virus Research Institute, Institute for Basic Science (IBS), Daejeon, Republic of Korea; 5 Department of Artificial Intelligence, Sungkyunkwan University, Suwon, Republic of Korea; 6 Graduate School of Health Science and Technology, Ulsan National Institute of Science and Technology (UNIST), Ulsan, Republic of Korea

**Keywords:** Monkeypox virus, AIM2, Innate immunity, Inflammation, Inflammasome, Inflammatory cell death, NOD-like receptors, Viral infection, Inflammasome

## Abstract

Monkeypox, a zoonotic disease caused by the monkeypox virus (MPXV), has significant global public health implications. Inflammasomes serve as crucial components of the innate immune system, detecting pathogens and triggering cell death in infected cells to eliminate harmful agents. However, the precise molecular mechanisms governing the activation of inflammasomes during MPXV infection remain largely unclear. Using CRISPR-knockout cytosolic innate immune sensor screening, we identified AIM2 as the sensor for MPXV within the inflammasome, a trigger for inflammatory cell death. Mechanistically, AIM2 forms a complex with essential cell death molecules, including ASC and caspase-1 (CASP1), without interacting with RIPK3 or CASP8. Loss of ASC, CASP1, or gasdermin D (GSDMD) reduced cell death following MPXV infection, whereas loss of GSDME, CASP3, CASP6, CASP7, CASP9, RIPK3, or MLKL did not. Pyroptotic cell death was predominantly observed in infected cells, whereas apoptotic and necroptotic signaling pathways were primarily activated in uninfected bystander cells. Furthermore, we found that the transcription factor IRF1 serves as an upstream regulator of AIM2, controlling AIM2-dependent cell death. In experiments involving AIM2-deficient mice infected with MPXV, we observed a decrease in proinflammatory cytokines, multiple inflammatory cell death pathways, and leukocyte migration, culminating in increased viral spread. CAST/EiJ mice succumbed to high-dose MPXV infection within 8 days, whereas AIM2 inhibition increased survival, with 10% of the mice treated with an AIM2 inhibitor surviving the infection. In a low-dose infection model, AIM2 inhibition reduced IL-1β and IL-18 production, LDH release, and tissue pathology. These findings highlight the critical role of AIM2-mediated inflammasome activation, along with multiple programmed cell death pathways, in shaping the innate immune response to MPXV infection, offering valuable insights for developing therapeutic strategies targeting AIM2 and the broader innate immune response against monkeypox.

## Introduction

Monkeypox is a zoonotic disease caused by the monkeypox virus (MPXV), with over 87,000 cases reported in more than 100 countries. This has led to hundreds of deaths and a significant strain on the global healthcare system [1]. The clinical outcomes of MPXV patients are strongly influenced by the innate immune response [2, 3]. Proper activation of this response and the resulting production of inflammatory cytokines are crucial for the natural antiviral immune reaction, which clears the virus and prevents prolonged infection. However, excessive activation can trigger cytokine storms, pathogenic inflammation, and tissue damage [2, 3].

Before cytokine release, several innate immune sensors, or pattern recognition receptors (PRRs), are responsible for detecting pathogens and inducing inflammatory signaling pathways and inflammatory cell death [4]. The major families of PRRs identified to date include membrane-bound Toll-like receptors (TLRs) and C-type lectin receptors (CLRs), cytosolic nucleotide-binding oligomerization domain (NOD)-like receptors (NLRs), retinoic acid-inducible gene-I (RIG-I)-like receptors (RLRs), and absent in melanoma 2 (AIM2)-like receptors (ALRs). Each sensor recognizes pathogen-associated or damage-associated molecular patterns (PAMPs or DAMPs, respectively) to activate related innate immune signaling pathways.

The critical role of the innate immune response is further demonstrated through inflammasome activation [5]. In the presence of PAMPs and DAMPs, inflammasome sensors form complex structures involving multiple proteins. This complex activates caspase-1 (CASP1), which subsequently cleaves gasdermin D (GSDMD), leading to cell death as a host defense mechanism to eliminate infected cells. This process plays a pivotal role in the release of inflammasome-dependent proinflammatory cytokines, specifically IL-1β and IL-18.

Numerous inflammasome sensors and their respective triggers have been extensively studied. NLRP3 responds to disturbances in cellular homeostasis, NLRC4 recognizes bacterial flagellin and type III secretion system components, AIM2 detects double-stranded DNA, and Pyrin is activated by the inhibition of Rho-GTPase activity [6]. Although vaccinia virus (VACV) infection has been reported to activate inflammasomes within the Poxviridae family [7, 8], the unique characteristics of MPXV necessitate identifying its specific inflammasome activation pathway to better understand its pathogenic mechanisms and immune evasion strategies, particularly given the significant public health impact of the virus.

In this study, we aimed to elucidate the role of inflammasome activation in the response to MPXV and its implications for inflammatory cell death. We provide the first genetic evidence that AIM2 functions as a sensor for MPXV and elucidate both the upstream and downstream molecular mechanisms of AIM2 during MPXV infection. Through CRISPR-mediated knockout of the cytosolic innate immune sensor, we identified AIM2 as a critical trigger for inflammatory responses associated with cell death. Mechanistically, AIM2 forms a complex with essential cell death molecules, including ASC and CASP1, but does not interact with RIPK3 or CASP8. Our results indicate that the loss of ASC, CASP1, or GSDMD significantly reduces cell death following MPXV infection, whereas the absence of GSDME, CASP3, CASP6, CASP7, CASP9, RIPK3, or MLKL does not impact cell death. The observed cell death was characterized by the activation of pyroptotic molecules in infected cells, as opposed to the activation of apoptotic and necroptotic markers in uninfected cells. Furthermore, our findings revealed that the transcription factor IRF1 functions as an upstream regulator of AIM2, modulating AIM2-dependent cell death. In experiments using AIM2-deficient mice infected with MPXV, we noted a decrease in proinflammatory cytokine production, impaired activation of multiple inflammatory cell death pathways, and reduced leukocyte migration—all of which led to increased viral spread. More importantly, CAST/EiJ mice succumbed to MPXV infection within 8 days; however, AIM2 inhibition significantly prolonged survival, with 10% of treated mice surviving the infection. These findings underscore the pivotal role of AIM2-mediated inflammasome activation and various programmed cell death pathways in orchestrating the innate immune response to MPXV infection. Our study provides valuable insights into the mechanisms of AIM2 and its potential as a target for therapeutic strategies aimed at enhancing the innate immune response against monkeypox and related viral infections.

## Results

### Identification of AIM2 as an inflammasome sensor for the monkeypox virus

**Fig. 1:**
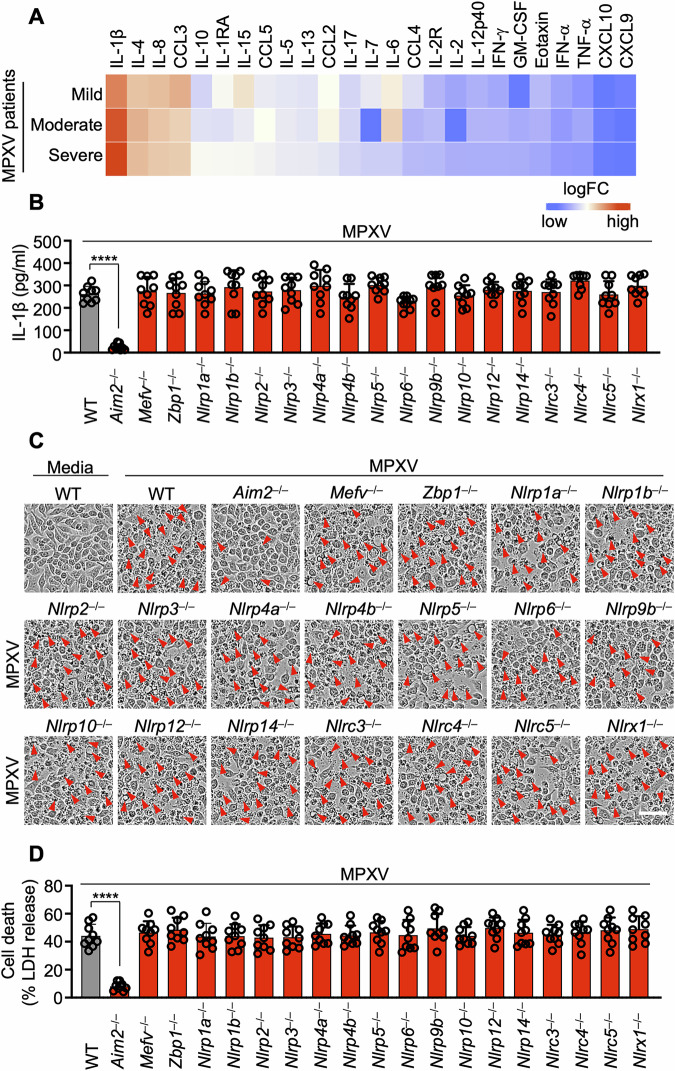
Identification of AIM2 as a cytosolic innate immune sensor for monkeypox virus. **A** Heatmap illustrating serum cytokine/chemokine concentration levels in patients with monkeypox infection. The values for monkeypox virus (MPXV) patients were acquired from previously published cytokine assays [2], which categorize patients on the basis of disease severity. Normal values were sourced from the Bio-Plex^®^ suspension array system tech note 6029 in Bio-Rad. The fold change was calculated for each disease severity compared with normal values as the baseline, represented on a logarithmic scale. Cytokines were ordered by the fold change under “severe” conditions. **B**–**D** Assessment of IL-1β release (**B**), cell death (**C**), and lactate dehydrogenase (LDH) release (**D**) in wild-type (WT) or the indicated cytosolic innate immune sensor-deficient immortalized bone marrow-derived macrophages (iBMDMs) following MPXV infection (MOI of 0.1 for 24 h). **B** and **D** Data are presented as the means ± s.e.m. ****P < 0.0001 (one-way ANOVA with Dunnett’s multiple comparisons test; *n* = 9 from 3 biologically independent samples). (**C**) shows images representative of a minimum of three independent experiments. Scale bar: 50 μm

To validate which cytokines are elevated following monkeypox virus (MPXV) infection, we first analyzed a publicly accessible dataset containing circulating cytokine levels in the serum of patients with varying degrees of MPXV disease severity (mild, moderate, and severe) [2]. We detected notable upregulation of circulating IL-1β, IL-4, IL-8, and CCL3 in correlation with disease severity (Fig. 1A). In particular, the level of IL-1β, an inflammasome-mediated cytokine, substantially increased with increasing severity of MPXV disease (Fig. 1A). This finding implicates inflammasome activation, as evidenced by elevated IL-1β levels, as a key driver of the inflammatory response during MPXV infection.

To determine the role of MPXV in inflammasome activation, we infected wild-type (WT) and caspase-1 (CASP1)-deficient primary bone marrow-derived macrophages (pBMDMs) with MPXV. Our findings indicated that MPXV infection promoted CASP1-dependent cleavage of CASP1 (Fig. S1A) and the release of the inflammasome-driven cytokines IL-1β (Fig. S1B) and IL-18 (Fig. S1C), suggesting that MPXV is involved in inflammasome activation. To precisely identify the cytosolic innate immune sensors essential for IL-1β production and cell death during MPXV infection, we conducted a comprehensive screen using a cytosolic innate immune sensor knockout library in murine immortalized bone marrow-derived macrophages (iBMDMs). The results of the screen revealed that AIM2 functions as an inflammasome sensor during MPXV infection (Fig. 1B–D). In contrast, other cytosolic innate immune sensors, including NLRP1a, NLRP1b, NLRP2, NLRP3, NLRP4a, NLRP4b, NLRP5, NLRP6, NLRP9b, NLRP10, NLRP12, NLRP14, NLRC3, NLRC4, NLRC5, NLRX1, Pyrin, and ZBP1, were found to have no role in IL-1β release or cell death in response to MPXV infection (Fig. 1B–D).

**Fig. 2:**
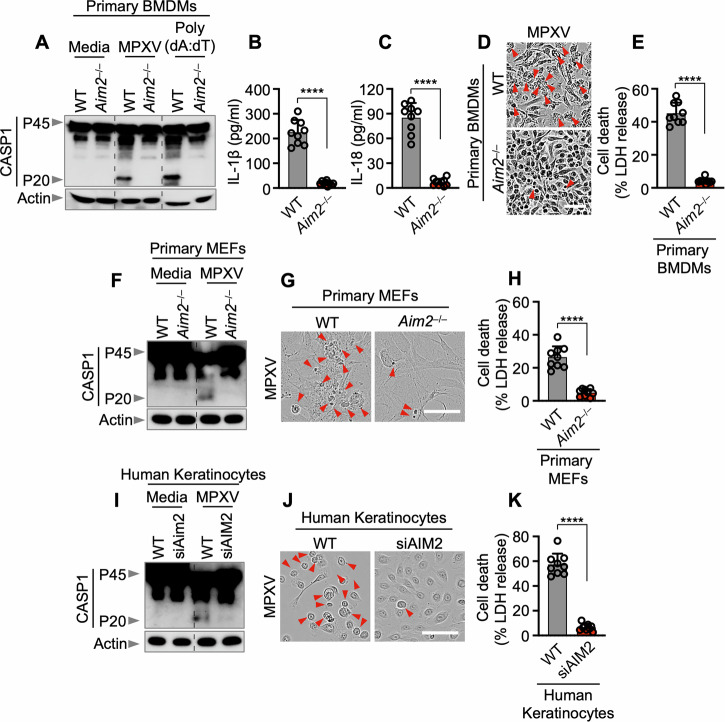
AIM2 is a pivotal cytosolic innate immune sensor that drives inflammasome activation and cell death in response to MPXV infection. **A** Immunoblot analysis of pro-caspase-1 (CASP1; P45) and cleaved CASP1 (P20) in monkeypox virus (MPXV)-infected or poly(dA:dT)-transfected wild-type (WT) or *Aim2*^–/–^ bone marrow-derived macrophages (BMDMs). **B**–**E** Assessment of IL-1β release (**B**) and IL-18 release (**C**) following MPXV infection (MOI of 0.1 for 24 h). **D** Cell death evaluation in BMDMs after MPXV infection. **E** Lactate dehydrogenase (LDH) release assessment after MPXV infection. **F**–**H** Immunoblot analysis of CASP1 (**F**), cell death images (**G**), and LDH release (**H**) from WT or *Aim2*^–/–^ mouse ear fibroblasts (MEFs) after MPXV infection (MOI of 0.1 for 24 h). **I**–**K** Immunoblot analysis of CASP1 (**I**), cell death images (**J**), and LDH release (**K**) from WT or AIM2-knockdown human keratinocytes (HEK001) by short-interfering RNA (siRNA) after MPXV infection (MOI of 0.1 for 24 h). (**A**), (**F**), (**I**) represent data from at least three independent experiments. **B**, **C**, **E**, **H**, **K** Data are presented as the means ± s.e.m. “ns” not significant, ****P < 0.0001 (two-tailed *t*-test; *n* = 9 from 3 biologically independent samples). (**D**), (**G**), and (**J**) show images representative of a minimum of three independent experiments. Scale bar: 50 μm

To determine whether the AIM2 inflammasome is crucial for host defense against MPXV, we infected WT and AIM2-deficient pBMDMs with MPXV. MPXV infection led to AIM2-dependent CASP1 cleavage, the release of the inflammasome-dependent cytokines IL-1β and IL-18, and cell death (Fig. 2A–E). Similarly, we also observed activation of CASP1 and cell death in primary mouse ear fibroblasts (MEFs) and human keratinocytes (HEK001) during MPXV infection, and this activation was inhibited by knockout of AIM2 or short-interfering RNA (siRNA)-mediated knockdown of AIM2 (Fig. 2F–K). These findings suggest that AIM2-dependent cell death also occurs in other primary murine cells and in human cells that, while not the natural host, are highly susceptible to MPXV infection. Conversely, no differences in CASP1 cleavage, IL-1β or IL-18 production, or cell death were noted between *Nlrp3*^–/–^ and *Nlrc4*^–/–^ pBMDMs and WT pBMDMs (Fig. S2A–J), suggesting that MPXV infection does not activate the NLRP3 or NLRC4 inflammasome. Notably, while infection with herpes simplex virus 1 (HSV1), a DNA virus, can trigger AIM2, leading to the cooperation of Pyrin and ZBP1 to facilitate AIM2 inflammasome activation and cell death [9], this mechanism was not observed during MPXV infection. There was no reduction in CASP1 cleavage, IL-1β, or IL-18 release, or cell death in *Mefv*^–/–^ and *Zbp1*^–/–^ pBMDMs (Fig. S2K–T). This conclusion was further supported by data from *Nlrp3*^–^^/^^–^ MEFs, which exhibited levels of cell death similar to those of WT controls following MPXV infection (Fig. S2U, V). Although NLRP3 is a well-characterized inflammasome sensor activated by diverse pathogens, our findings indicate that it is dispensable for inflammasome activation during MPXV infection. This observation is consistent with previous reports showing that DNA viruses such as HSV1 primarily engage in inflammasome signaling via AIM2, Pyrin, and ZBP1 rather than via NLRP3 [9, 10]. Collectively, these results establish AIM2 as the principal cytosolic innate immune sensor that mediates inflammasome activation and inflammatory cell death in response to MPXV infection.

### AIM2 drives inflammatory cell death in response to monkeypox virus infection

**Fig. 3:**
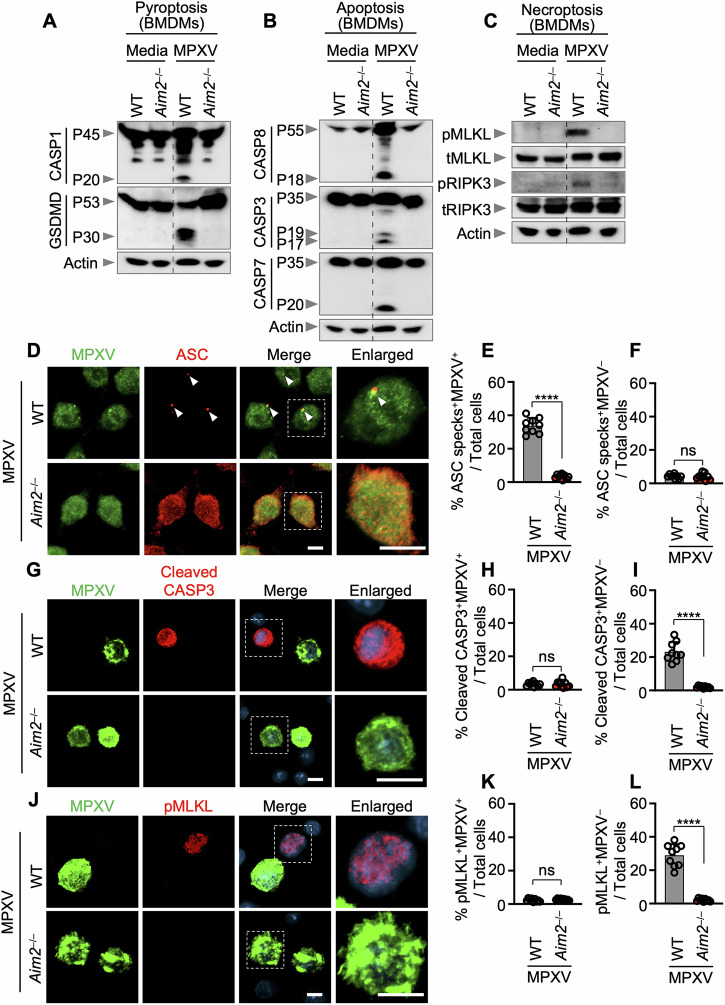
AIM2 drives inflammatory cell death in response to monkeypox virus infection. **A**–**C** Immunoblot analysis of pro-caspase-1 (CASP1; P45) and activated CASP1 (P20), pro-gasdermin D (GSDMD) (P53) and activated GSDMD (P30) (**A**); pro-CASP8 (P55) and cleaved CASP8 (P18), pro-CASP3 (P35) and cleaved CASP3 (P17/P19), pro-CASP7 (P35) and cleaved CASP7 (P20) (**B**); phosphorylated MLKL (pMLKL), total MLKL (tMLKL), phosphorylated RIPK3 (pRIPK3), and total RIPK3 (tRIPK3) (**C**) in bone marrow-derived macrophages (BMDMs) from WT or *Aim2*^−/−^ BMDMs after monkeypox virus (MPXV) infection (MOI of 0.1 for 24 h). **D** Immunofluorescence (IF) images of WT or *Aim2*^−/−^ BMDMs stained with MPXV and ASC at 24 h after MPXV infection (MOI of 0.1). Scale bars: 5 μm. Arrowheads indicate ASC specks. **E** Quantification of the percentage of ASC specks^+^MPXV^+^ cells among total cells. **F** Quantification of the percentage of cells with ASC specks^+^MPXV^−^ cells among total cells. **G** Immunofluorescence images of WT or *Aim2*^−/−^ BMDMs stained with MPXV and cleaved caspase-3 at 24 h after MPXV infection (MOI of 0.1). Scale bars: 5 μm. **H** Quantification of the percentage of cleaved caspase-3 (CASP3)^+^MPXV^+^ cells among total cells. **I** Quantification of the percentage of cleaved CASP3^+^MPXV^−^ cells among total cells. **J** Immunofluorescence images of WT or *Aim2*^−/−^ BMDMs stained with MPXV and pMLKL at 24 h after MPXV infection (MOI of 0.1). Scale bars: 5 μm. **K** Quantification of the percentage of pMLKL^+^MPXV^+^ cells among total cells. **L** Quantification of the percentage of pMLKL^+^MPXV^−^ cells among total cells. (**A**), (**B**), (**C**) represent data from at least three independent experiments. (**D**), (**G**), and (**J**) show images representative of a minimum of three independent experiments. Scale bar: 5 μm (merge), 6 μm (enlarged). **E**, **F**, **H**, **I**, **K**, **L** Data are presented as the means ± s.e.m. “ns” not significant, ****P < 0.0001 (two-tailed *t*-test; *n* = 9 from 3 biologically independent samples)

In addition to inflammasome activation, numerous pathogens and sterile triggers have been shown to induce a range of programmed cell death pathways (PCDs), including pyroptosis, apoptosis, and necroptosis [9, 11–23]. Therefore, our objective was to perform a biochemical characterization of MPXV-induced cell death. In WT pBMDMs, MPXV infection activated crucial molecules associated with the pyroptosis, apoptosis, and necroptosis pathways (Fig. 3A–C). However, in *Aim2*^–/–^ pBMDMs, there was a complete absence of activation of pyroptotic molecules, including CASP1 and gasdermin D (GSDMD), upon MPXV infection (Fig. 3A). Additionally, there was no cleavage of apoptotic CASP8, CASP3, or CASP7 in *Aim2*^–/–^ pBMDMs following MPXV infection (Fig. 3B). Furthermore, robust phosphorylation of the necroptotic molecules MLKL and RIPK3 did not occur in *Aim2*^–/–^ pBMDMs upon MPXV infection (Fig. 3C). These findings collectively suggest that the induction of multiple types of inflammatory PCD during MPXV infection is dependent on AIM2.

As MPXV infection induced the activation of multiple cell death molecules, including CASP1, CASP3, CASP7, and CASP8, GSDMD, RIPK3, and MLKL (Fig. 3A–C), we sought to understand the relative contribution of each of these molecules to cell death via a genetic approach. Compared with WT iBMDMs, *Asc*^*−/−*^, *Casp1*^*−/−*^, and *Gsdmd*^*−/−*^ but not *Gsdme*^*−/−*^ iBMDMs resulted in reduced cell death (Fig. S3A, B). In contrast, compared with WT iBMDMs, *Casp3*^*−/−*^, *Casp6*^*−/−*^, *Casp7*^*−/−*^, *Casp9*^*−/−*^, *Ripk3*^*−/−*^, and *Mlkl*^*−/−*^ iBMDMs presented similar dynamics of cell death (Fig. S3C–F), indicating that pyroptotic effectors, specifically ASC, CASP1, and GSDMD, play a central role in MPXV-induced cell death.

To evaluate differences in viral infection efficiency between pyroptotic and apoptotic/necroptotic cell death, we conducted immunofluorescence staining using antibodies against MPXV (viral infection marker) alongside ASC (pyroptosis marker), cleaved CASP3 (apoptosis marker), or phosphorylated MLKL (pMLKL) (necroptosis marker). Although MPXV infectivity was similar between WT and *Aim2*^*–/–*^ pBMDMs, ASC speck formation occurred in an AIM2-dependent manner, predominantly in infected cells (Figs. 3D–F and S3G). In contrast, the population of cleaved CASP3-positive and phosphorylated MLKL-positive cells included bystander WT pBMDMs, although apoptotic/necroptotic cell death was also AIM2 dependent (Fig. 3G–L). These findings indicate that AIM2-dependent pyroptosis occurs in infected cells, whereas apoptosis and necroptosis are associated with bystander cells.

### The AIM2 inflammasome mediates inflammatory cell death upon monkeypox virus infection

**Fig. 4:**
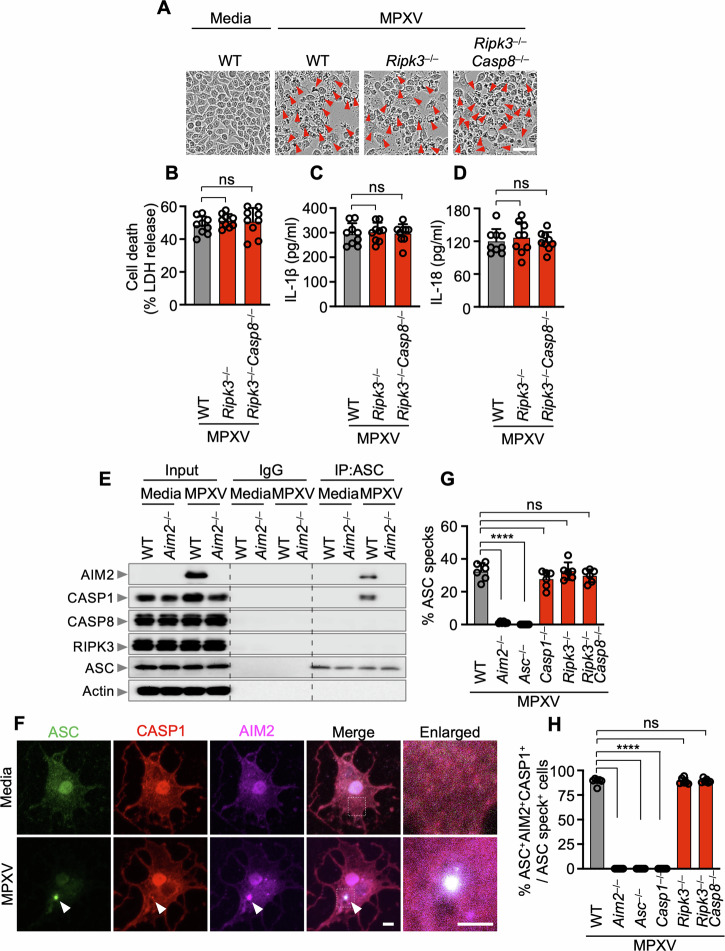
The CASP8-RIPK3 axis is not needed for the assembly of the AIM2 inflammasome in response to monkeypox virus infection. **A** Cell death assessment in WT, *Ripk3*^–/–^, or *Ripk3*^−/−^*Casp8*^−/−^ immortalized bone marrow-derived macrophages (iBMDMs) after monkeypox virus (MPXV) infection (MOI of 0.1 for 24 h). **B**–**D** Evaluation of lactate dehydrogenase (LDH) release (**B**), IL-1β release (**C**), and IL-18 release (**D**) following MPXV infection (MOI of 0.1 for 24 h). **E** Immunoprecipitation (IP) of WT and *Aim2*^−/−^ BMDMs with either IgG control antibodies or anti-ASC antibodies after MPXV infection. **F** Immunofluorescence (IF) images of WT iBMDMs at 24 h after MPXV infection (MOI of 0.1). Arrowheads indicate ASC specks. **G** Quantification of the percentage of cells with ASC specks among total cells. **H** Quantification of the percentage of cells with ASC^+^AIM2^+^CASP1^+^ specks among *ASC* speck^+^ cells. (**A**), (**F**) show images representative of a minimum of three independent experiments. Scale bar: 50 μm (**A**), 5 μm (**F**). **B**–**D**, **G**, **H** Data are presented as the means ± s.e.m. “ns” not significant, ****P < 0.0001 (one-way ANOVA with Dunnett’s multiple comparisons test; *n* = 9 from 3 biologically independent samples). **E** Data from at least three independent experiments are presented

To scrutinize the role of other cell death proteins in this context, we employed a genetic model and validated these findings via *Ripk3*^–/–^*Casp8*^–/–^ iBMDMs, in which the key cell death components involved in apoptosis and necroptosis are absent. Although the CASP8/RIPK3 axis drives various inflammatory PCDs during live-pathogen infections, such as HSV1 [9], *Francisella novicida* [9], influenza A virus [12, 13], murine hepatitis virus [14, 24], and PAMPs/DAMPs [25, 26], there was no significant discrepancy in cell death, IL-1β, or IL-18 release among WT, *Ripk3*^–/–^, and *Ripk3*^–/–^*Casp8*^–/–^ iBMDMs upon MPXV infection (Fig. 4A–D). Through immunoprecipitation post-MPXV infection, we noted that ASCs interact with AIM2 and CASP1 but not with CASP8 or RIPK3; these interactions were not detected in *Aim2*^–/–^ BMDMs (Fig. 4E). To further confirm the formation of this multiprotein complex, we observed the colocalization of *ASC* specks with AIM2 and CASP1 within the same cell at 24 h post-MPXV infection (Fig. 4F). Notably, this pattern persisted in *Ripk3*^–/–^ and *Ripk3*^–/–^*Casp8*^–/–^ iBMDMs (Fig. 4G, H), indicating the presence of AIM2 within this multiprotein complex, along with ASC and CASP1, during MPXV infection.

### IRF1 regulates inflammatory cell death and type I IFN–inducible immune effectors during MPXV infection

**Fig. 5:**
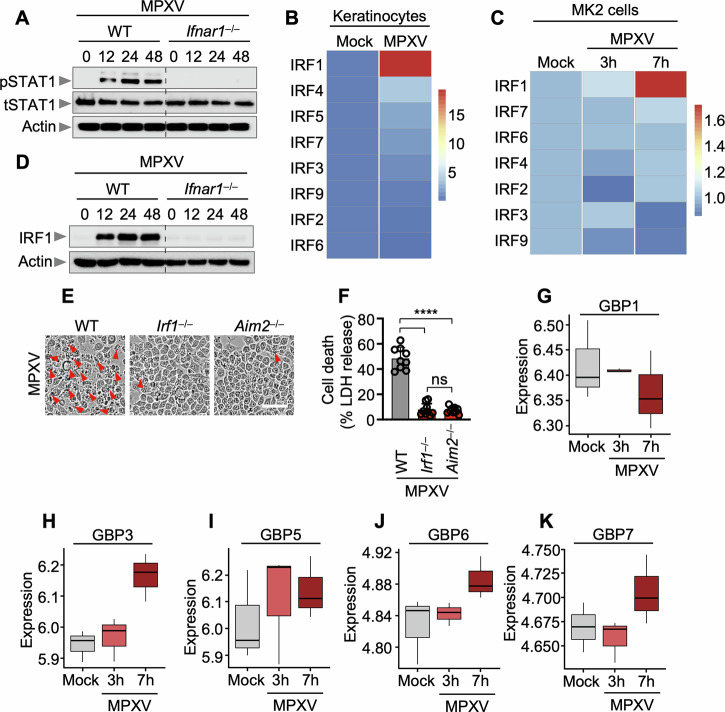
The transcription factor IRF1 acts upstream of AIM2 and regulates cell death. **A** Immunoblot analysis of phosphorylated STAT1 (pSTAT1) and total STAT1 (tSTAT1) in wild-type (WT) and *Ifnar1*^–^^/^^–^ bone marrow-derived macrophages (BMDMs) over a defined time course following monkeypox virus (MPXV) infection (MOI of 0.1). **B**, **C** Heatmaps comparing relative gene expression between control (mock-treated) and MPXV-infected cells. **B** Average expression levels of interferon regulatory factor (IRF) family genes in MPXV-infected human keratinocytes relative to mock controls [29]. **C** Temporal expression patterns of IRF family genes in MPXV-infected MK2 cells [30]. **D** Immunoblot analysis of IRF1 expression in WT and *Ifnar1*^–^^/^^–^ BMDMs following MPXV infection (MOI of 0.1) over time. Quantification of cell death (**E**) and lactate dehydrogenase (LDH) release (**F**) in WT, *Irf1*^–^^/^^–^, and *Aim2*^–^^/^^–^ immortalized BMDMs (iBMDMs) after MPXV infection (MOI of 0.1, 24 h). Boxplots depicting the average expression levels of guanylate-binding proteins (GBPs), including GBP1 (**G**), GBP3 (**H**), GBP5 (**I**), GBP6 (**J**), and GBP7 (**K**), at multiple time points in MPXV-infected MK2 cells [30]. GBP2 and GBP4 were excluded because of the absence of corresponding probes. Boxes represent the median and interquartile range (IQR); whiskers indicate 1.5× IQR. (**A**) and (**D**) show representative data from at least three independent experiments. (**E**) presents representative images from a minimum of three independent experiments (scale bar, 50 μm). (**F**) shows data presented as the mean ± s.e.m.; “ns” not significant, ****P < 0.0001 (one-way ANOVA with Dunnett’s multiple comparisons test; *n* = 9 from three biologically independent samples)

To elucidate the upstream regulatory mechanisms of AIM2-mediated cell death during MPXV infection, we investigated the involvement of type I interferon (IFN) signaling. The phosphorylation of STAT1 (pSTAT1), a canonical downstream effector of type I IFN signaling, was markedly reduced in *Ifnar1*^–^^/^^–^ pBMDMs following MPXV infection (Fig. 5A), confirming the effective disruption of IFNAR1-dependent signaling in these cells. These findings support a functional role for type I IFN signaling in coordinating innate immune responses to MPXV. Given prior reports linking interferon regulatory factors (IRFs) to inflammasome signaling [27, 28], we examined the potential contribution of IRFs to MPXV pathogenesis. Reanalysis of publicly available transcriptomic datasets from MPXV-infected human keratinocytes and monkey kidney epithelial cells (MK2 Cells) [29, 30] revealed that IRF1 was the most highly upregulated IRF in both cell types (Fig. 5B, C). Moreover, IRF1 expression was diminished in *Ifnar1*^–^^/^^–^ pBMDMs following MPXV infection (Fig. 5D), indicating that IRF1 functions downstream of type I IFN signaling. Compared with wild-type controls, *Irf1*^–^^/^^–^ iBMDMs consistently exhibited significantly reduced cell death (Fig. 5E, F). Transcriptomic profiling of MPXV-infected MK2 cells at 7 h post-infection further revealed the upregulation of multiple guanylate-binding protein (GBP) family members, including GBP3, GBP5, GBP6, and GBP7, highlighting a broader antiviral gene program downstream of type I IFN–IRF1 signaling (Fig. 5G–K). Collectively, these findings identify IRF1 as a key transcriptional regulator of type I IFN–mediated innate immune responses, integrating inflammasome activation and GBP-driven antiviral defenses during MPXV infection.

### AIM2 drives the inflammatory response and monkeypox pathogenesis

To translate the implications of AIM2-dependent multiple types of inflammatory programmed cell death observed in vitro to an in vivo context, we extended our investigation to a mouse model of MPXV infection. A dose-dependent increase in MPXV protein expression within the infected lungs of WT mice (C57BL/6J) was evident at 7 days post-infection (Fig. S4A). Furthermore, the IL-1β, IL-18, and LDH levels in bronchoalveolar lavage fluid (BALF) from WT mice increased in a dose-dependent manner 7 days after MPXV infection (Fig. S4B–D). However, the TNF-α and IFN-γ levels remained comparable to those in the PBS-treated group (Fig. S4E, F). These observations underscore the potential of MPXV infection to stimulate viral and proinflammatory cytokine release in WT mice (C57BL/6J).

**Fig. 6:**
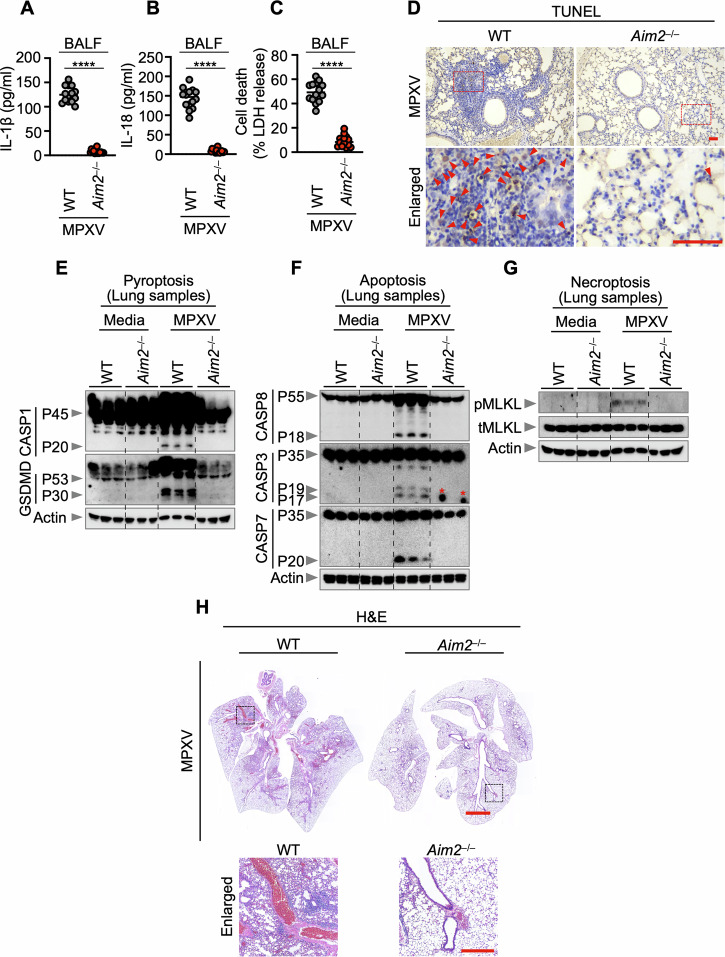
AIM2 regulates inflammatory responses, viral spread, and inflammation in response to the monkeypox virus. **A**–**C** Quantification of IL-1β release (**A**), IL-18 release (**B**), and lactate dehydrogenase (LDH) release (**C**) in bronchoalveolar lavage fluid (BALF) from WT (*n* = 14) and *Aim2*^−/−^ (*n* = 14) mice on day 5 following monkeypox virus (MPXV) (western Africa strain) infection (5 × 10^4^ PFU). **D** TUNEL staining of lung tissue from MPXV (western Africa strain)-infected WT and *Aim2*^−/−^ mice at 5 days. TUNEL-positive cells are shown in red. **E**–**G** Immunoblot analysis of pro-caspase-1 (CASP1) and activated CASP1 (P45 and P20, respectively), pro-gasdermin D (GSDMD) (P53) and activated GSDMD (P30) (**E**), pro-caspase-8 (CASP8) and cleaved caspase-8 (P55 and P18, respectively), pro-caspase-3 (CASP3) and cleaved CASP3 (P35, P19, and P17, respectively), pro-caspase-7 (CASP7) and cleaved CASP7 (P35 and P20, respectively) (**F**), and phosphorylated MLKL (pMLKL) and total MLKL (tMLKL) (**G**) in lung samples extracted from WT or *Aim2*^−/−^ mice at 5 days post-MPXV (Western Africa strain) infection (5 × 10^4^ PFU). The red asterisk signifies a nonspecific band. **H** Hematoxylin and eosin (H&E) staining of lung tissue from MPXV (western Africa strain)-infected WT and *Aim2*^−/−^ mice (5 × 10^4^ PFU). For (**A**–**C**), each symbol represents an individual mouse. The data from two independent experiments were combined. The data are expressed as the means ± s.e.m. ****P < 0.0001 (two-tailed *t*-test). For (**D**), scale bars are 0.1 mm. Images are representative of five independent experiments. For (**E**–**G**), each lane represents an independent biological replicate. For (**H**), scale bars are 2 mm (entire lung field) and 0.5 mm (enlarged field). Images are representative of five independent experiments

IL-1β, IL-18, and LDH levels in the BALF 5 days after MPXV infection exhibited an AIM2-dependent effect (Fig. 6A–C). This reduction correlated with reduced cell death, as indicated by a diminished number of terminal deoxynucleotidyl transferase-mediated deoxyuridine triphosphate nick end labeling (TUNEL)-positive cells in the lungs of *Aim2*^−/−^ mice compared with those in the lungs of infected WT mice (Fig. 6D), potentially contributing to extensive necrosis in the latter. Consistent with the TUNEL results, *Aim2*^−/−^ lung tissues presented decreased activation of CASP1, GSDMD, CASP8, CASP3, and CASP7, as well as pMLKL, in response to MPXV compared with WT lung tissues (Fig. 6E–G). Histological scrutiny of lung slices from WT mice revealed bronchiolitis characterized by reduced alveolar air space and leukocyte infiltration, whereas lung slices from *Aim2*^–/–^ mice exhibited a milder inflammatory response on day 7 after MPXV infection (Fig. 6H).

To evaluate the role of AIM2 in controlling MPXV replication and spread, we conducted quantitative viral genome analyses via qPCR in both in vitro and in vivo models. In *Aim2*^–^^/^^–^ iBMDMs, compared with WT control cells, MPXV-infected cells presented significantly elevated viral DNA levels at 24 h post-infection (Fig. S5A). Similarly, lungs from *Aim2*^–^^/^^–^ mice presented higher MPXV genome copy numbers than those from WT mice did (Fig. S5B). Compared with those of WT mice, the lung tissues of Aim2^–/–^ mice presented increased MPXV protein expression following MPXV infection (Fig. S5C). Moreover, increased viral dissemination was evident in *Aim2*^–/–^ mice compared with WT mice, as substantiated by significantly amplified MPXV viral staining in their lungs at 7 days post-infection (Fig. S5D). These results indicate that AIM2 limits MPXV replication both in vitro and in vivo.

**Fig. 7:**
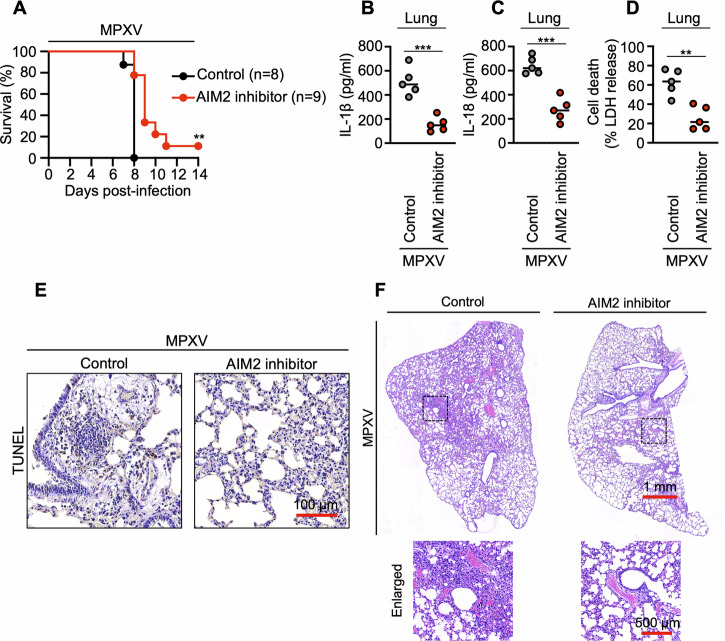
AIM2 inhibition reduces MPXV-induced mortality, inflammation, and tissue pathology in CAST/EiJ mice. **A** Survival of CAST/EiJ mice following high-dose intranasal challenge with monkeypox virus (MPXV, Central Africa strain; 1 × 10^5^ PFU). The mice were intraperitoneally administered an AIM2 inhibitor (ODN TTAGGG sodium, 0.3 mg per dose) or vehicle control (DPBS) at 6 h post-infection and again on days 1, 2, and 3. **B**–**C** Quantification of IL-1β (**B**) and IL-18 (**C**) levels in lung homogenates collected on day 3 post-infection (5 × 10⁴ PFU), as measured via ELISA. **D** Lactate dehydrogenase (LDH) release in lung homogenates as a marker of cell death (5 × 10⁴ PFU). **E** Representative images of TUNEL staining of lung tissue sections on day 3 post-infection (5 × 10⁴ PFU). **F** Hematoxylin and eosin (H&E) staining of lung sections showing inflammatory cell infiltration (5 × 10⁴ PFU). In (**A**), **P < 0.01 (log-rank [Mantel–Cox] test). In (**B**–**D**), each symbol represents an individual mouse; data are pooled from two independent experiments and presented as the mean ± s.e.m. ***P < 0.001, **P < 0.01 (two-tailed *t*-test). (**E**) and (**F**) show representative images from five independent experiments

To assess the therapeutic potential of pharmacological AIM2 inhibition during MPXV infection, we employed the CAST/EiJ mouse model, which is highly susceptible to Clade I (Central African strain) MPXV and exhibits rapid disease progression and lethality [31], unlike C57BL/6J mice, which do not develop fatal outcomes under similar conditions. Following high-dose infection (10^5^ PFU), untreated CAST/EiJ mice died within 8 days, whereas AIM2 inhibitor treatment (ODN TTAGGG sodium) significantly prolonged survival, with 10% of treated mice surviving the infection (Fig. 7A). Under a lower infectious dose (5 × 10⁴ PFU), AIM2 inhibition markedly reduced the levels of inflammatory cytokines, including IL-1β and IL-18 (Fig. 7B, C), indicating that AIM2 is involved in the cytokine storm associated with MPXV pathogenesis. LDH release was also significantly decreased in inhibitor-treated mice (Fig. 7D), indicating reduced cell death. This finding was supported by a lower number of TUNEL-positive cells in the lung tissue (Fig. 7E). Histopathological evaluation (H&E staining) further revealed diminished leukocyte infiltration and attenuated inflammation in the lungs of AIM2 inhibitor-treated mice compared with vehicle-treated controls (Fig. 7F). Collectively, these results demonstrate that pharmacological inhibition of AIM2 attenuates MPXV-induced inflammatory responses, tissue injury, and mortality in susceptible hosts.

## Discussion

Monkeypox, caused by the Monkeypox virus (MPXV), has become a major global health concern, resulting in a significant number of reported cases and fatalities. This infection triggers a proinflammatory response, recruiting immune cells such as macrophages and neutrophils to combat the virus. Identifying the innate immune sensors responsible for inflammatory cell death and cytokine release is crucial for the defense of host against MPXV infection. However, excessive activation can lead to cytokine storms, pathogenic inflammation, and tissue damage. Nonetheless, the specific innate immune sensors involved in MPXV infection remain largely unknown. In this study, we present the first genetic evidence demonstrating that AIM2 acts as an MPXV sensor, driving inflammatory cell death and promoting the production of inflammatory cytokines.

Although MPXV is best known for its cutaneous manifestations, it can also be transmitted via respiratory droplets and cause pulmonary infection, particularly in animal models [1, 32, 33]. In this study, we employed an intranasal infection model to investigate AIM2-dependent inflammatory responses and the systemic pathogenesis of MPXV. However, the role of AIM2 within the skin microenvironment, where MPXV induces primary lesions, remains undefined. As a cytosolic DNA sensor, AIM2 may modulate local immune responses in cutaneous tissues, potentially influencing both antiviral defense and inflammation-mediated tissue damage.

AIM2 is a DNA sensor that has been extensively studied across various pathogens, including bacteria [8, 10, 34] and viruses [8, 9]. When confronted with PAMPs and DAMPs, AIM2 forms a complex that triggers caspase-1 (CASP1) activation, resulting in inflammatory signaling and cell death. Emerging data suggest that AIM2, in conjunction with other inflammasome sensors, can be activated in response to live-pathogen infections due to the presence of multiple PAMPs and DAMPs [6, 9, 35]. Previously, we demonstrated that the inflammasome sensor AIM2 orchestrates the innate immune sensors Pyrin and ZBP1, steering inflammatory signaling and inflammatory cell death and offering host protection during herpes simplex virus 1 (HSV1) and *Francisella novicida* infections [9]. Notably, AIM2 was the only innate immune sensor activated in response to MPXV infection, whereas a broad panel of other inflammasome-associated receptors showed no detectable activation. Further investigations are necessary to explore the potential synergy between AIM2 and other innate immune sensors in response to MPXV infection.

The functional relevance of AIM2 extends beyond cytokine release to the regulation of programmed cell death pathways (PCDs). Our study demonstrated that AIM2 activation orchestrates pyroptosis, which is the primary mechanism of cell death in MPXV-infected cells and is mediated by CASP1 and gasdermin D (GSDMD). Additionally, AIM2 indirectly contributes to apoptosis and necroptosis in bystander cells, highlighting its role in coordinating diverse PCD pathways to support viral replication. Notably, unlike other live-pathogen infections [9, 12–14, 24–26], while these secondary cell death pathways are AIM2 dependent, they are not mediated through direct interactions with apoptosis- and necroptosis-related molecules, such as CASP8 or RIPK3, suggesting a broader network of signaling interactions downstream of AIM2 activation. This spatial and mechanistic differentiation between cell death pathways provides new insights into the cellular dynamics of AIM2-mediated MPXV pathogenesis.

Finally, in vivo therapeutic experiments demonstrated that oligonucleotide-mediated inhibition of AIM2 in CAST/EiJ mice increased survival and attenuated proinflammatory cytokine production, cell death, and tissue damage following MPXV infection. These findings underscore the pathological role of AIM2 in vivo and support its potential as a therapeutic target for severe MPXV infection.

The urgency and significance of our study lie in the imperative to devise efficacious therapeutic interventions against this emerging infectious malady. Given the substantial morbidity and mortality rates associated with MPXV infections, unraveling the host immune response and pinpointing potential therapeutic targets are of paramount importance. By revealing the role of AIM2 and its associated inflammatory PCDs, we provide valuable insights into the nuanced mechanisms underlying the immune response to MPXV infection. Therapeutic targeting of AIM2 and its downstream inflammatory pathways represents a promising strategy for mitigating monkeypox pathogenesis and increasing pandemic preparedness.

## Methods

### Mice

C57BL/6J wild-type (WT) mice were obtained from Hyochang Science. *Aim2*^–^^/^^–^ and *Casp1*^–^^/^^–^ mice (both on a C57BL/6J background), as well as CAST/EiJ mice, were purchased from Jackson Laboratory. *Nlrp3*^–^^/^^–^, *Nlrc4*^–^^/^^–^, *Mefv*^–^^/^^–^, and *Zbp1*^–^^/^^–^ mice (on a C57BL/6J background) were procured from Cyagen. The mice were group-housed, with up to five mice per cage, and bred under standard pathogen-free conditions in the animal facility at the Ulsan National Institute of Science and Technology (UNIST). The mice were maintained on a 12-h light/dark cycle (lights on from 7 AM to 7 PM) and provided with standard chow. Both male and female mice were included in this study. In vivo investigations utilized age- and sex-matched mice aged 6–8 weeks, whereas in vitro studies involved mice aged 6–12 weeks. Cohoused animals were chosen for in vivo analyses. All experimental procedures were executed following protocols approved by the Institutional Animal Care and Utilization Committee of UNIST.

## Cell culture

Primary bone marrow-derived macrophages (BMDMs) were cultured for six days in Iscove’s modified Dulbecco’s medium (IMDM, Thermo Fisher Scientific, Cat. No. 12440061) supplemented with 10% fetal bovine serum (FBS, Thermo Fisher Scientific, Cat. No. 16000044), 30% L929-conditioned medium, 1% nonessential amino acids (Thermo Fisher Scientific, Cat. No. 11140-050), and 1% penicillin‒streptomycin solution (Thermo Fisher Scientific, Cat. No. 15070-063). These BMDMs were seeded in 12-well plates at a density of 1 million cells/well and incubated overnight before further use. L929 cells (ATCC, CCL-1) were procured and cultured in IMDM supplemented with 10% FBS (Thermo Fisher Scientific, Cat. No. 16000044), 1% nonessential amino acids, and 1% penicillin‒streptomycin. Immortalized bone marrow-derived macrophages (iBMDMs), a generous gift from Dr. Tae-Hyuk Kwon (UNIST), were cultivated in Dulbecco’s modified Eagle’s medium (DMEM, Thermo Fisher Scientific, Cat. No. 11995081) supplemented with 10% FBS and 1% penicillin‒streptomycin. HEK001 cells were procured and cultured in keratinocyte-serum-free medium (GIBCO-BRL 17005-042) supplemented with 5 ng/ml human recombinant EGF and 2 mM L-glutamine (without bovine pituitary extract or serum). Mouse ear fibroblasts (MEFs) were cultivated in Dulbecco’s modified Eagle’s medium (DMEM, Thermo Fisher Scientific, Cat. No. 11995081) supplemented with 10% FBS and 1% penicillin‒streptomycin.

### Generation of knockout clonal cells

WT and *Gsdmd*^−/−^ iBMDMs were generously provided by Tae-Hyun Kwon (UNIST). *Nlrp1a*^−/−^, *Nlrp1b*^−/−^, *Casp1*^−/−^, *Ripk3*^−/−^, and *Ripk3*^−/−^*Casp8*^−/−^ iBMDMs were generated previously [36]. In this study, we generated *Aim2*^−/−^, *Mefv*^−/−^, *Zbp1*^−/−^, *Nlrp2*^−/−^, *Nlrp3*^−/−^, *Nlrp4a*^−/−^, *Nlrp4b*^−/−^, *Nlrp5*^−/−^, *Nlrp6*^−/−^, *Nlrp9b*^−/−^, *Nlrp10*^−/−^, *Nlrp12*^−/−^, *Nlrp14*^−/−^, *Nlrc3*^−/−^, *Nlrc4*^−/−^, *Nlrc5*^−/−^, *Nlrx1*^−/−^, *Asc*^−/−^, *Gsdme*^−/−^, *Casp3*^−/−^, *Casp6*^−/−^, *Casp7*^−/−^, *Casp9*^−/−^, *Mlkl*^−/−^, and *Irf1*^−/−^ iBMDMs. The WT iBMDM cells underwent electroporation with 1.7 µg of Cas9 protein (IDT) and 5 µg of in vitro transcribed gRNA, following the Amaxa 4D-Nucleofector program DS-136. For electroporation, a total of 4 × 10^5^ cells were employed. To create single-cell-derived knockout clones in iBMDMs, the transfected cells were seeded at a low density (0.5 cells/well) in a 96-well plate. After a period of 10–14 days, each well was scrutinized for the presence of viable single-cell-derived clones. These clones were detached via trypsin, and genomic DNA extraction was carried out via the DNeasy Blood & Tissue Kit (Qiagen, 69504). To confirm the knockout of the target gene in the single-cell-derived clones, genomic regions encompassing the Cas9 target site were subjected to amplification via KAPA HiFi HotStart PCR polymerase (KK2502). The resulting amplicons were further amplified via TruSeq HT dual-index-containing primers to produce libraries for deep sequencing. The sequencing of these libraries was accomplished via an Illumina iSeq system equipped with a paired-end sequencing system. The calculation of mutation frequencies was performed via the MAUND program, which is accessible at https://github.com/ibs-cge/maund.

### Gene silencing mediated by siRNA

The siRNA against the *AIM2* gene was purchased from Life Technologies. The nucleotide sequences of the siRNAs used in this study were as follows: 5ʹ-GACAUCUGGAGUUCAUAGCACCAUA-3ʹ and 5ʹ-UAUGGUGCUAUGAACUCCAGAUGUC-3ʹ. For transfection, 15 pmol of siRNA was incubated with 500 μl of Opti-MEM and 7.5 μl of Lipofectamine RNAi Max (Life Technologies) for 15 min at room temperature. The mixture was transferred into 12-well plates at a density of 10^5^ human keratinocytes/well. As a negative control, nonspecific scrambled siRNA was used.

### Virus culture

The monkeypox virus (MPXV) of the Western Africa strain (clade IIb) was generously supplied by Marc-Alain Widdowson from the Institute of Tropical Medicine, Antwerp, Belgium. The MPXV strain of Central Africa (clade Ia) was isolated from a sample of an MPXV-infected person generously supplied by Steve AHUKA from the Institut National de Recherche Biomedicale (INRB), Democratic Republic of the Congo. The virus was cultivated in Vero cells (obtained from the American Type Culture Collection; CCL-81^TM^). The viral titer was determined via a plaque assay conducted on Vero cells. The influenza A virus (A/Puerto Rico/8/34, H1N1 (PR8)) was courteously provided by Dr. Man-Seong Park from Korea University, Republic of Korea. The virus was propagated in 11-day-old embryonated chicken eggs through allantoic inoculation. The titer of the influenza A virus was gauged via a plaque assay performed on MDCK cells (provided by Dr. Atsushi Kawaguchi from the University of Tsukuba, Japan).

### Cell stimulation and infection

To induce ligand-mediated AIM2 inflammasome activation, we transfected 1 ng of poly(dA:dT) (InvivoGen, tlrl-patn) via DOTAP (Roche, 11202375001) following the manufacturer’s instructions and then incubated the cells for 6 h. To activate the NLRP3 inflammasome, the cells were first primed for 4 h with 500 ng/ml ultrapure lipopolysaccharide (LPS) from *Escherichia coli* (0111:B4) (InvivoGen, tlrl-3pels) and subsequently stimulated for 6 h with 1 μg/ml nigericin (SIGMA, N7143). To trigger NLRC4 inflammasome activation, we transfected 10 ng of flagellin (InvivoGen, tlrl-epstfla-5) via DOTAP (Roche, 11202375001) following the manufacturer’s instructions and incubated the cells for 6 h. For Pyrin inflammasome activation, we transfected 0.5 μg/ml of TcdB (R&D systems, 6246-GT) via DOTAP (Roche, 11202375001) according to the manufacturer’s instructions, followed by a 6-h incubation period. In the context of MPXV clade IIb infection (Western Africa strain) (24 h, MOI of 0.1), the cells were infected with high-glucose DMEM (Gibco, 11995-065). Similarly, for IAV infection (12 h; MOI of 10), the cells were infected with high-glucose DMEM (WElGene, LM001-03).

### Cell death imaging and analysis

We employed an IncuCyte S3 imaging system (Sartorius) for this study. Cell death analysis was performed following established methodologies [9, 24, 37–40], utilizing the same IncuCyte S3 imaging system. BMDMs or iBMDMs were seeded into 12-well plates at a density of 10^6^ cells/well and subjected to stimulation. After the stipulated incubation period, SYTOX Green (Thermo Fisher Scientific, S7020) was administered following the manufacturer’s protocol. The resulting images were subjected to analysis via software packaged with the IncuCyte imager.

### Cytokine analysis

Cytokines were assessed via IL-1β ELISA (Invitrogen, BMS6002TEN), IL-18 ELISA (R&D Systems, DY7625-05), TNF-β ELISA (R&D Systems, MTA00B), IFN-β ELISA (R&D Systems, SMIF00), and an LDH cytotoxicity kit (Promega; G1780), following the guidelines provided by the respective manufacturers.

### Immunoblotting analysis

Immunoblotting was conducted following established protocols [9, 24, 37, 39, 41]. To assess caspase (CASP) activity, the cells were lysed along with their supernatant with 50 μl of caspase lysis buffer (comprising 1× protease inhibitors, 1× phosphatase inhibitors, 10% NP-40, and 25 mM DTT), followed by the addition of 100 μl of 4× SDS loading buffer. For signaling analysis, cell supernatants were collected at specified time points, and after one wash with PBS, the cells were lysed with RIPA buffer. Proteins were resolved through electrophoresis on 8–15% polyacrylamide gels. After electrophoretic transfer onto PVDF membranes (Millipore, IPVH00010), nonspecific binding was blocked with 5% skim milk. The membranes were subsequently incubated with the following primary antibodies: anti-caspase-1 (AdipoGen, AG-20B-0042, 1:2000), anti-caspase-3 (CST, #9662, 1:2000), anti-cleaved caspase-3 (CST, #9661, 1:2000), anti-caspase-7 (CST, #9492, 1:2000), anti-cleaved caspase-7 (CST, #9491, 1:2000), anti-caspase-8 (CST, #4927, 1:2000), anti-cleaved caspase-8 (CST, #8592, 1:2000), anti-pMLKL (CST, #37333, 1:2000), anti-MLKL (Abgent, AP14272b, 1:2000), anti-GSDMD (Abcam, ab209845, 1:2000), anti-pRIPK3 (CST, 91702 S, 1:2000), anti-RIPK3 (ProSci, 2283, 1:2000), anti-IRF1 (CST, 8478, 1:2000 dilution), anti-pSTAT1 (CST, 7649, 1:2000 dilution), and anti-tSTAT1 (CST). The anti-VACV antibody is a polyclonal reagent that detects conserved orthopoxvirus antigens, including late-stage MPXV structural proteins. After washing, the membranes were exposed to suitable horseradish peroxidase (HRP)-conjugated secondary antibodies (diluted 1:5000; Jackson ImmunoResearch Laboratories, anti-rabbit [111-035-047], and anti-mouse [315-035-047]) for 1 h. The protein bands were visualized via Luminata Forte Western HRP Substrate (Millipore, WBLUF0500), and the membranes were examined via Amersham ImageQuant 800 UV. The resulting images were analyzed via ImageJ software (v1.54).

### Immunofluorescence staining

Immunofluorescence staining was conducted as previously described [9, 42–45]. In brief, the cells were fixed with 4% paraformaldehyde (PFA) for 10 min, permeabilized with PBS containing 0.5% Triton X-100 for 3 min, and subsequently incubated in PBS containing 1% skim milk for 1 h. For MPXV-ASC staining, the coverslips were incubated with anti-ASC (Millipore, 04-147) and anti-Vaccinia (also detects MPXV; Santa Cruz, sc-58210) antibodies. The secondary antibodies used were Alexa Fluor 488-conjugated anti-mouse IgG (Life Technologies, A21202; 1:200) and Alexa Fluor 568-conjugated anti-rabbit IgG (Life Technologies, A10042; 1:200). For MPXV-cleaved caspase-3 or MPXV-pMLKL staining, the coverslips were incubated with anti-Vaccinia (also known as MPXV; Abcam, ab35219) and either anti-cleaved caspase-3 (CST, #9661) or anti-pMLKL (Abcam, ab196436) antibodies. For AIM2-ASC-caspase-1 staining, Alexa Fluor 488 (Thermo Fisher Scientific, A20181) was employed for labeling anti-ASC (Millipore, 04-147), Alexa Fluor 568 (Thermo Fisher Scientific, A20184) was utilized for anti-caspase-1 conjugation (AdipoGen, AG-20B-0042), and Alexa Fluor 647 (Thermo Fisher Scientific, A20186) was employed for anti-AIM2 conjugation (Abcam, ab119791) in accordance with the manufacturer’s instructions. The coverslips were subjected to a 1-h incubation with the specified antibodies (1:100). For visualization, DAPI mounting medium (P36931; Invitrogen, Carlsbad, CA, USA) was used for counterstaining. The images were captured via a confocal laser scanning microscope (LSM900; Carl Zeiss) equipped with a 63× apochromatic objective.

### Immunoprecipitation

Immunoprecipitation was carried out following previously established protocols [9, 39, 43]. In brief, subsequent to MPXV infection, the cells were lysed in buffer comprising 20 mM Tris-HCl (pH 7.4), 100 mM NaCl, 30 mM KCl, and 0.1% NP-40. Following centrifugation at 16,000 × *g* for 10 min, the lysates were subjected to an overnight incubation at 4 °C with either an IgG control antibody (CST, 3900 S) or an anti-ASC antibody (AdipoGen; AG-25B-006-C100), along with protein A/G PLUS-Agarose (Santa Cruz Biotechnology, sc-2003). Following subsequent washing with the aforementioned buffer, the immunoprecipitated proteins were eluted with 0.1 mM glycine (pH 3.0).

### Quantitative PCR for MPXV genome detection

Quantitative PCR (qPCR) was used to determine monkeypox virus (MPXV) genome levels in infected tissues and cells via the CFX Duet Real-Time PCR System (Bio-Rad). Total DNA was extracted from formalin-fixed lung tissues (25–50 mg) or infected immortalized bone marrow-derived macrophages (iBMDMs) via the DNeasy Blood & Tissue Kit (Qiagen, Cat. No. 69504) following the manufacturer’s protocol. A SYBR Green–based qPCR assay targeting the *F3L* gene, a highly conserved region of the MPXV genome, was employed for viral quantification. The primer sequences were as follows: forward (MPXV-F3L-F), 5′-CACACCGTCTCTTCCACAGA-3′; reverse (MPXV-F3L-R), 5′-GATACAGGTTAATTTCCACATCG-3′. Reactions were performed in a 20 μL volume using iTaq™ Universal SYBR® Green Supermix (Bio-Rad, Cat. No. 1725120), consisting of 10 μL of SYBR Green Supermix, 300 nM of each primer, and 100 ng of template DNA. All reactions were run in technical triplicate, with no-template controls (NTCs) included to monitor for contamination. To normalize the input DNA and assess sample quality, mouse *β-actin* was amplified in parallel via the following primers: forward, 5′-GGCTGTATTCCCCTCCATCG-3′; reverse, 5′-CCAGTTGGTAACAATGCCATGT-3′.

### In vivo infection

Age- and sex-matched cohorts of 6–8-week-old WT and *Aim2*^–^^/^^–^ mice (C57BL/6J background) or CAST/EiJ mice cohoused under identical conditions were used for the MPXV infection experiments. The protocol for MPXV infection was approved by the Korea National Institute of Health IACUC (protocols KDCA-IACUC-23-007 and KDCA-IACUC-24-028). All MPXV-related procedures were conducted under animal biosafety level 3 conditions at the Korea National Institute of Health. These procedures adhered strictly to the safety protocols and operational guidelines of the Korea National Institute of Health. The mice were anesthetized via a combination of 100 mg/kg ketamine and 10 mg/kg Rompun before intranasal infection. WT and *Aim2*^–/–^ mice were infected with MPXV (Western Africa strain) in 50 μl of PBS solution containing approximately 5 × 10^4^ PFU plaque-forming units (PFUs). Lungs harvested on either day 5 or day 10 post-infection were homogenized in 1 ml of RIPA buffer for subsequent immunoblot analysis. For the assessment of cytokine levels, bronchoalveolar lavage fluid (BALF) was collected from the lungs of the specified mice on days 5 or 10 following infection. For CAST/EiJ mice, MPXV (Central Africa strain) was administered intranasally in 20 μl of PBS containing either 10^5^ PFU (for survival analysis) or 5 × 10⁴ PFU (for cytokine and histopathological assessment). On days 1, 2, and 3 post-infection, the mice received intraperitoneal injections of 0.3 mg of AIM2 inhibitor (ODN TTAGGG sodium; MCE, Cat# HY-150751C) diluted in DPBS.

### Histology and immunohistochemical staining

Hematoxylin and eosin (H&E) staining, MPXV immunohistochemistry, and TUNEL staining were conducted through JIREH BioPath Co., Ltd. Lung tissue sections, with a thickness of 4 μM, were embedded in paraffin and subjected to H&E, MPXV (Abcam, ab35219), and TUNEL staining, following the manufacturer’s guidelines. The samples were examined via a DMi8 inverted microscope (Leica).

### Analysis of serum cytokine levels in monkeypox patients

We acquired the serum concentrations of cytokines and chemokines in patients infected with MPXV from a previously published study [2]. Serum cytokine and chemokine concentrations were derived from the average of triplicate measurements via the Cytokine Human Magnetic 30-Plex Panel. In this study, patients were categorized on the basis of geographical location, source of infection, and disease severity, as classified by the World Health Organization scoring system for smallpox [46, 47]. In parallel, the serum cytokine and chemokine concentrations of uninfected individuals were sourced from Bio-Rad’s Bio-Plex^®^ suspension array system tech note 6029 (www.Bio-Radad.com). The mean concentration within this range was selected as the baseline for cytokine and chemokine serum concentrations in healthy individuals. We subsequently visualized the serum cytokine and chemokine levels via heatmaps generated in R (ComplexHeatmap, v.2.14.0). For each disease state, we computed the fold change, using the normal values as the reference baseline. These fold-change values were logarithmically transformed with a base of 10. To organize the presentation, the cytokines featured in the heatmap were ordered in descending order according to the fold change between serum concentrations under severe and normal conditions.

### Visualization of RNA expression in MPXV-infected cell lines

We obtained RNA expression data for MPXV-infected cell lines from previously published studies [29, 30]. To compare the RNA expression levels between control (mock) cell lines and MPXV-infected cell lines, we generated heatmaps. In the study by Watanabe et al., three samples of keratinocytes were used for both mock and MPXV infection. MPXV strains have been grouped into three clades: the Congo Basin clade (clade Ia), the West Africa clade (clade IIb), and 2022 MPXV (clade IIb) [29]. Alkhalil et al. had three groups of MK2 cells with three samples each for the control, 3 h post-infection, and 7 h post-infection with the monkeypox virus-Katako Kombe strain (MPV-KK) from the Democratic Republic of the Congo [30]. For heatmap visualization, we selected the expression values for genes of the IRF family (IRF1 to IRF9). The expression values were first averaged across groups for each dataset. In particular, values from three clades from Watanabe et al. were all grouped as “infected.” Then, the average values were normalized by dividing the mean expression value of the control or mock samples. In the study by Watanabe et al., the expression values for IRF8 were omitted since all samples had a value of 0. Additionally, in the study by Alkhalil et al., IRF5 and IRF8 were omitted because there were no matching genes in the dataset. Heatmaps were then generated in R via the pheatmap package (v1.0.12). To visualize the expression of guanylate-binding proteins (GBP1–GBP7), we generated boxplots via the ggplot2 package (v3.5.1) and compared the three time points from the Alkhalil et al. dataset [30]. GBP2 and GBP4 were excluded from this analysis because of the absence of corresponding probes in the dataset.

### Statistical analysis

We performed data analysis via GraphPad Prism 10.0 software. The data are expressed as the mean ± SEM. We determined statistical significance via a two-tailed *t*-test, one-way ANOVA, or two-way ANOVA with multiple comparisons for analyses involving multiple groups. Survival analysis was performed via the log-rank (Mantel–Cox) test. Statistical significance was acknowledged for *P*-values less than 0.05 and denoted as *P < 0.05, **P < 0.01, ***P < 0.001, and ****P < 0.0001.

## Materials availability

All unique reagents generated in this study are available from the lead contact.

## Supplementary information

uncropped WB

Supplementary Figure 1

Supplementary Figure 2

Supplementary Figure 3

Supplementary Figure 4

Supplementary Figure 5

Supplementary Figure Legends

## Data Availability

All the data needed to evaluate the conclusions in the paper are presented in the paper or the Supplementary Materials. This manuscript encompasses all analyses, and the datasets sourced from publicly available repositories are detailed in the study (specifically, serum cytokine levels from MPXV patients [2] and RNA expression data from MPXV-infected cell lines [29, 30]). Materials referenced in this study can be acquired from the corresponding author upon reasonable request.

## Code availability

All the data needed to evaluate the conclusions in the paper are presented in the paper or the Supplementary Materials. This manuscript encompasses all analyses, and the datasets sourced from publicly available repositories are detailed in the study (specifically, serum cytokine levels from MPXV patients [2] and RNA expression data from MPXV-infected cell lines [29, 30]). Materials referenced in this study can be acquired from the corresponding author upon reasonable request.
