# Supplementary material for: AIM2 drives inflammatory cell death and monkeypox pathogenesis
Source: Cell Mol Immunol. 2025 Nov 12;22(12):1615–28. doi: 10.1038/s41423-025-01367-7 (PMC12661009; doi:10.1038/s41423-025-01367-7)
Supplement: Supplementary file 1 — uncropped WB [file 41423_2025_1367_MOESM1_ESM.pdf]

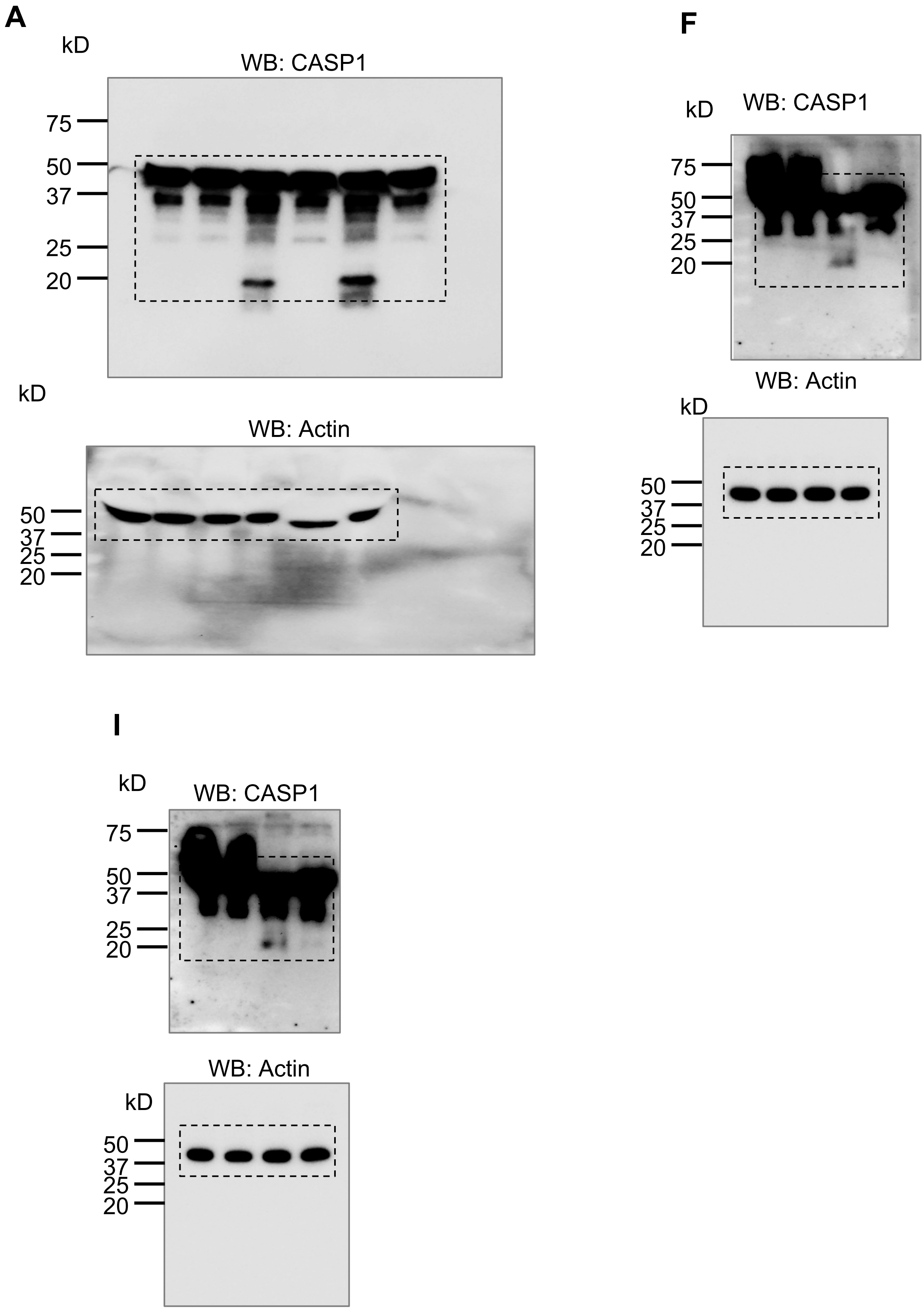
A
kD
WB: CASP1
75
50
37
25
20
kD
WB: Actin
50
37
25
20
F
kD
WB: CASP1
75
50
37
25
20
WB: Actin
kD
50
37
25
20
I
kD
WB: CASP1
75
50
37
25
20
WB: Actin
kD
50
37
25
20

Figure 2

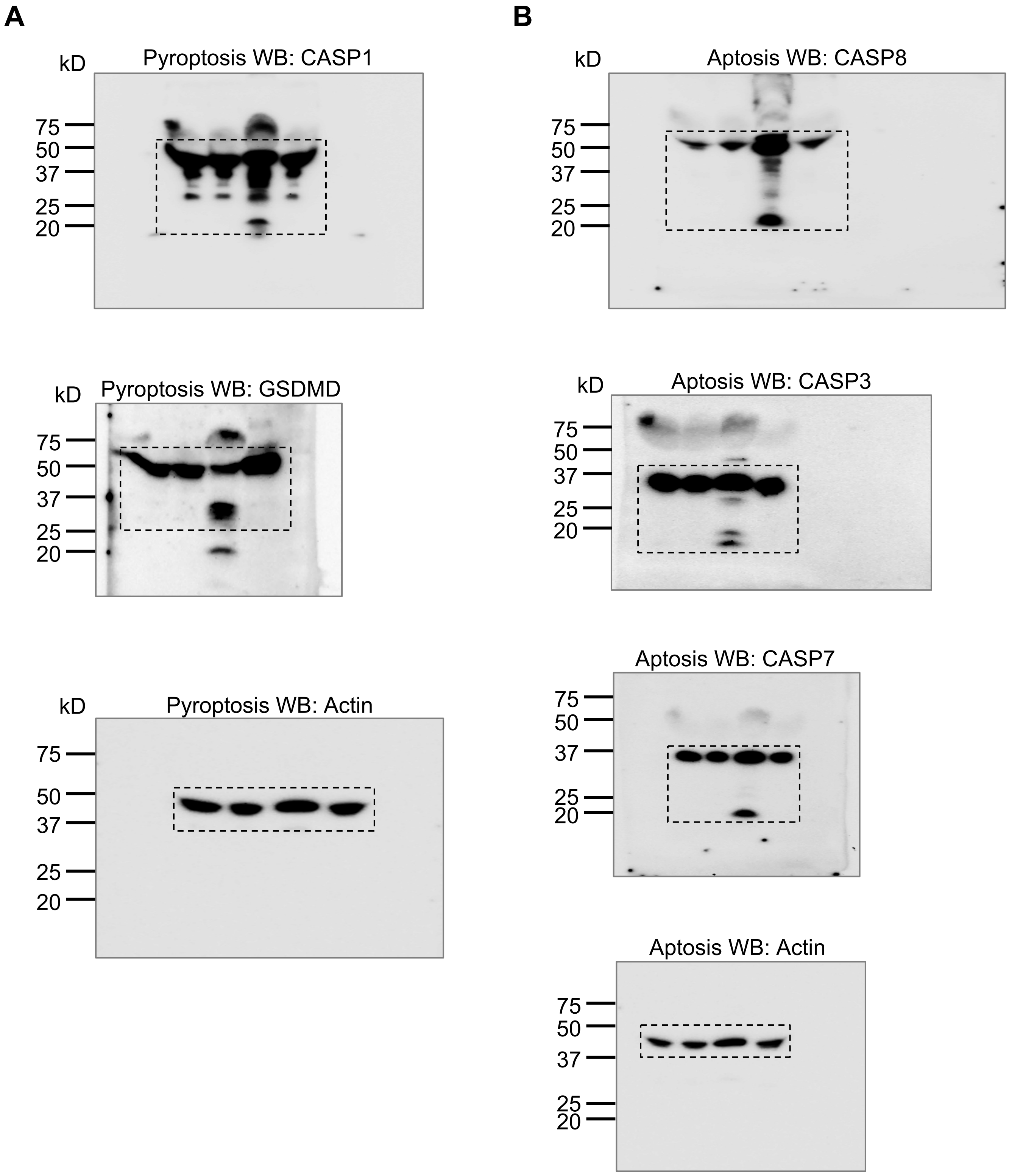
A
B
Pyroptosis WB: CASP1
kD
75
50
37
25
20
Pyroptosis WB: GSDMD
kD
75
50
37
25
20
Pyroptosis WB: Actin
kD
75
50
37
25
20
Aptosis WB: CASP8
kD
75
50
37
25
20
Aptosis WB: CASP3
kD
75
50
37
25
20
Aptosis WB: CASP7
75
50
37
25
20
Aptosis WB: Actin
75
50
37
25
20

Figure 3

C

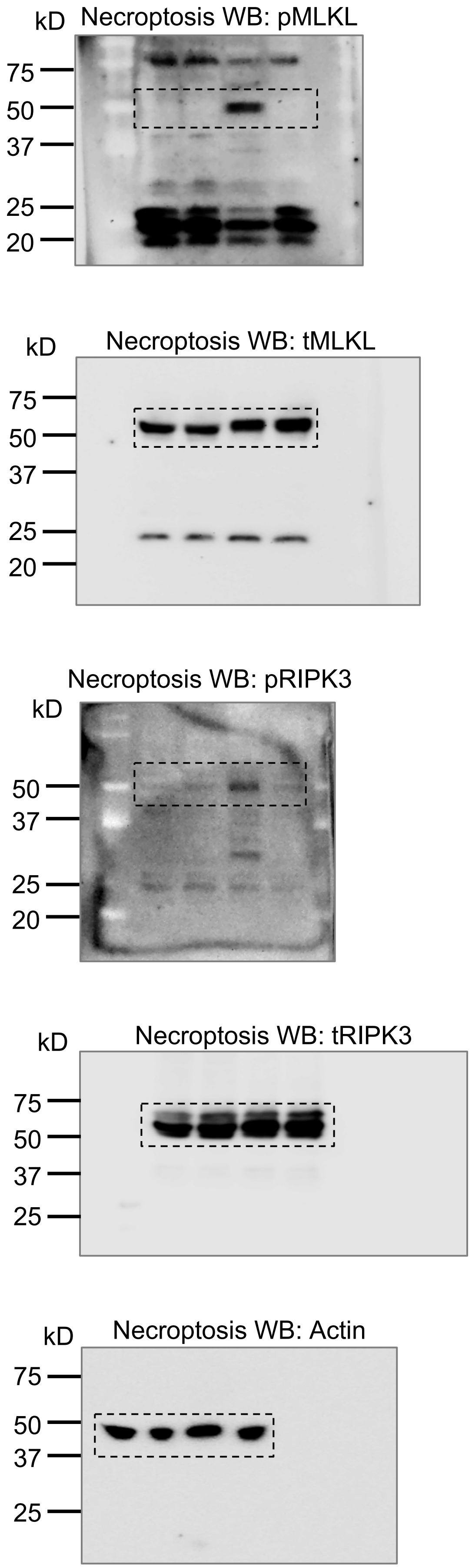

kD
Necroptosis WB: pMLKL
75
50
37
25
20
kD
Necroptosis WB: tMLKL
75
50
37
25
20
Necroptosis WB: pRIPK3
kD
50
37
25
20
kD
Necroptosis WB: tRIPK3
75
50
37
25
kD
Necroptosis WB: Actin
75
50
37
25

Figure 3

E

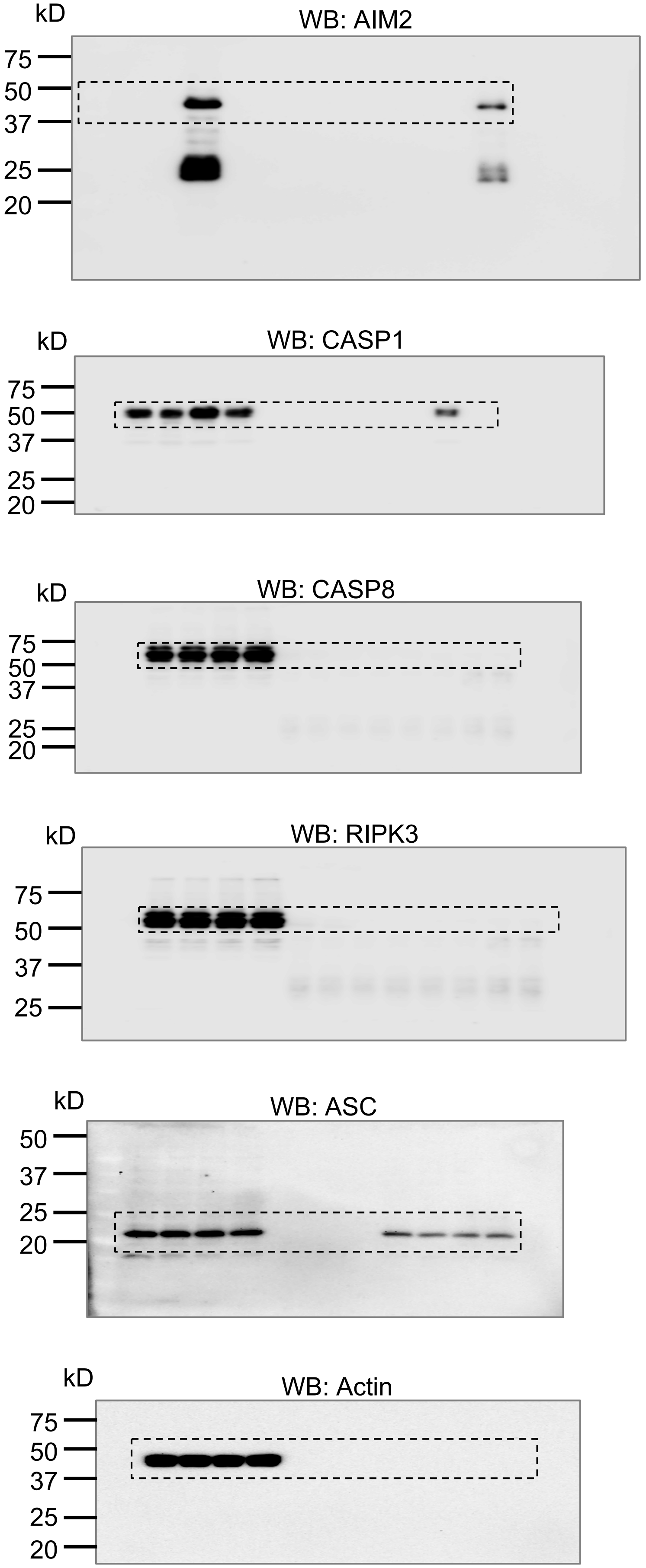
kD
WB: AIM2
75
50
37
25
20
kD
WB: CASP1
75
50
37
25
20
kD
WB: CASP8
75
50
37
25
20
kD
WB: RIPK3
75
50
37
25
kD
WB: ASC
50
37
25
20
kD
WB: Actin
75
50
37
25
20

Figure 4

A

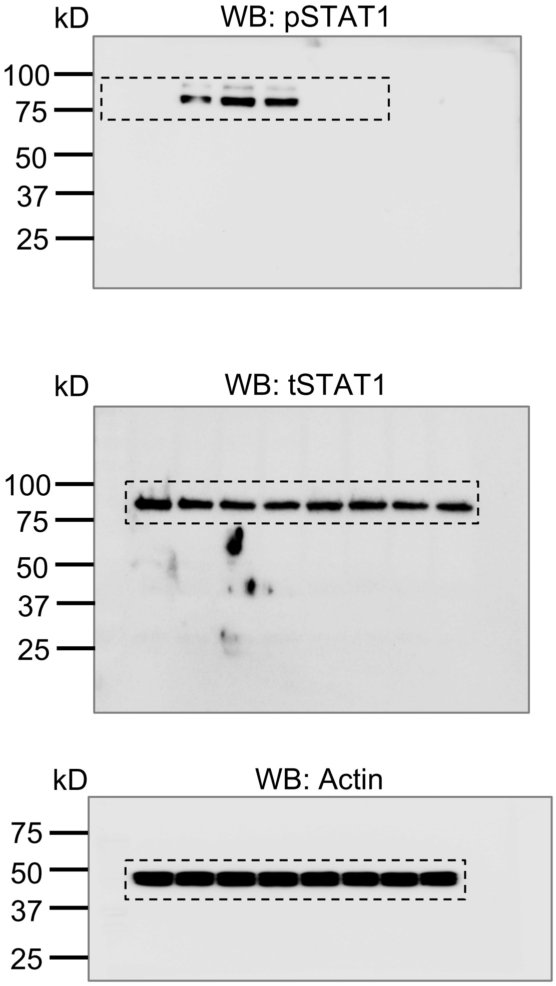
kD
WB: pSTAT1
100
75
50
37
25
kD
WB: tSTAT1
100
75
50
37
25
kD
WB: Actin
75
50
37
25

D

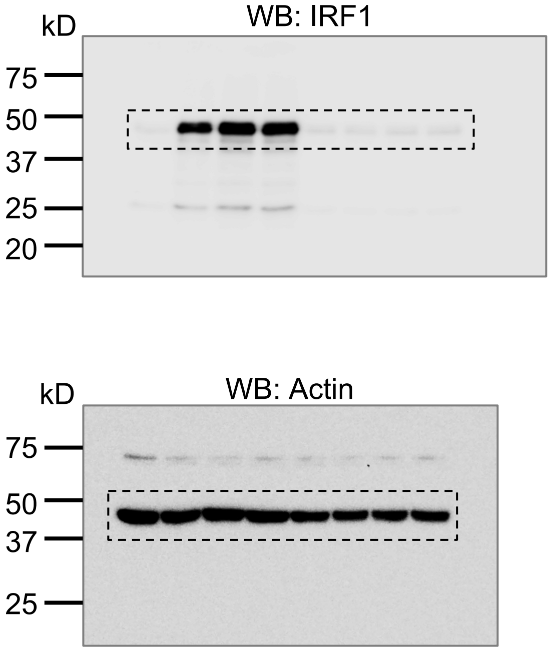
kD
WB: IRF1
75
50
37
25
20
kD
WB: Actin
75
50
37
25

Figure 5

E

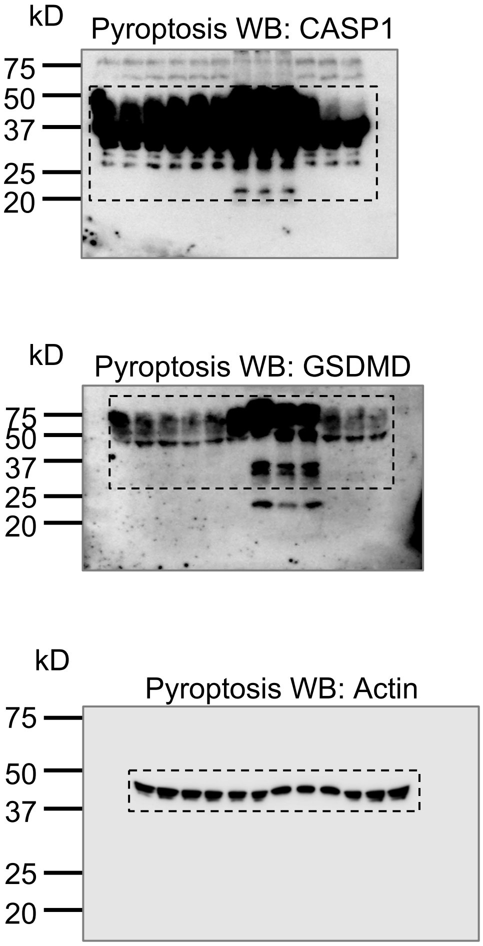
kD
Pyroptosis WB: CASP1
75
50
37
25
20
kD
Pyroptosis WB: GSDMD
75
50
37
25
20
kD
Pyroptosis WB: Actin
75
50
37
25
20

F

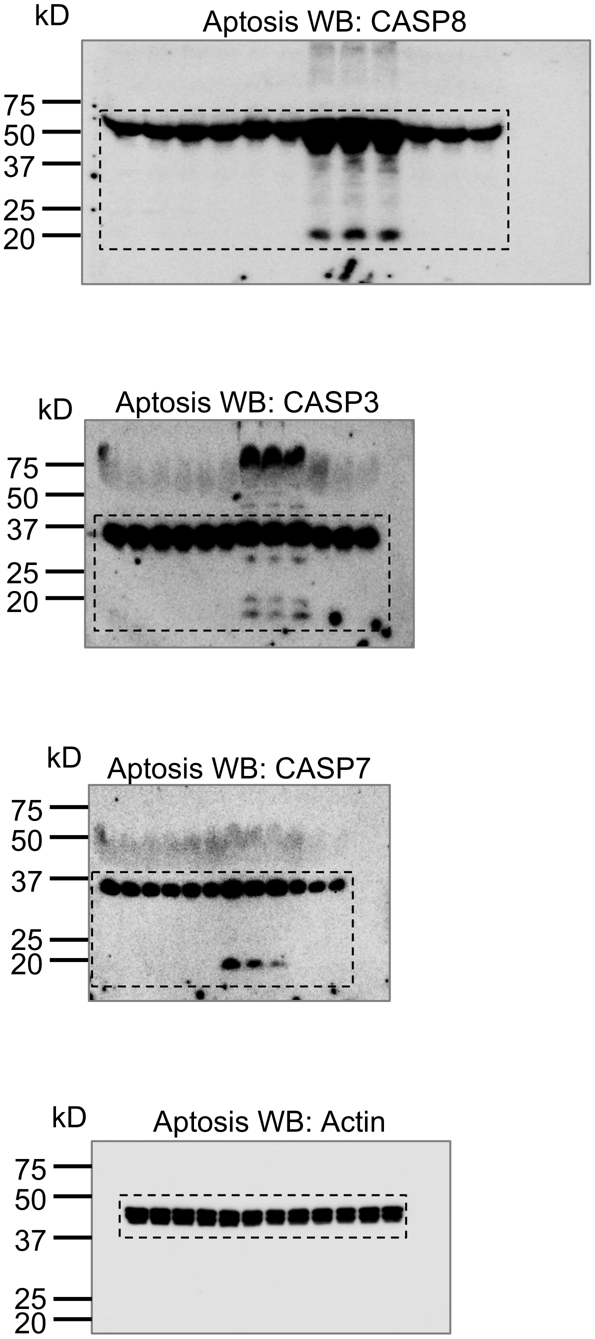
kD
Aptosis WB: CASP8
75
50
37
25
20
kD
Aptosis WB: CASP3
75
50
37
25
20
kD
Aptosis WB: CASP7
75
50
37
25
20
kD
Aptosis WB: Actin
75
50
37
25
20

Figure 6

G

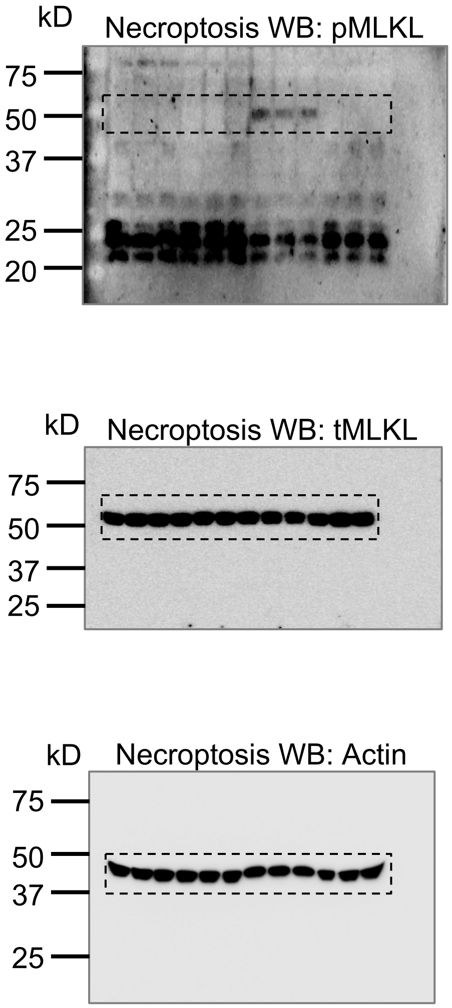
kD
Necroptosis WB: pMLKL
75
50
37
25
20
kD
Necroptosis WB: tMLKL
75
50
37
25
kD
Necroptosis WB: Actin
75
50
37
25

Figure 6

A

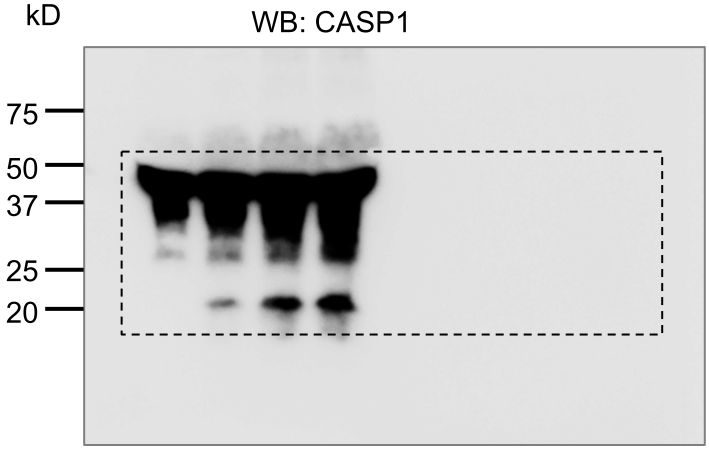

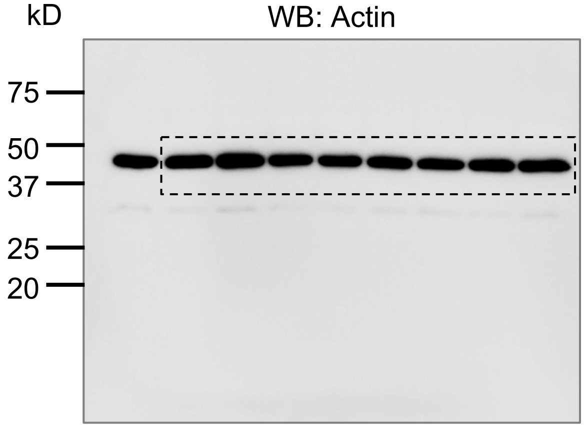

Supplementary Figure 1

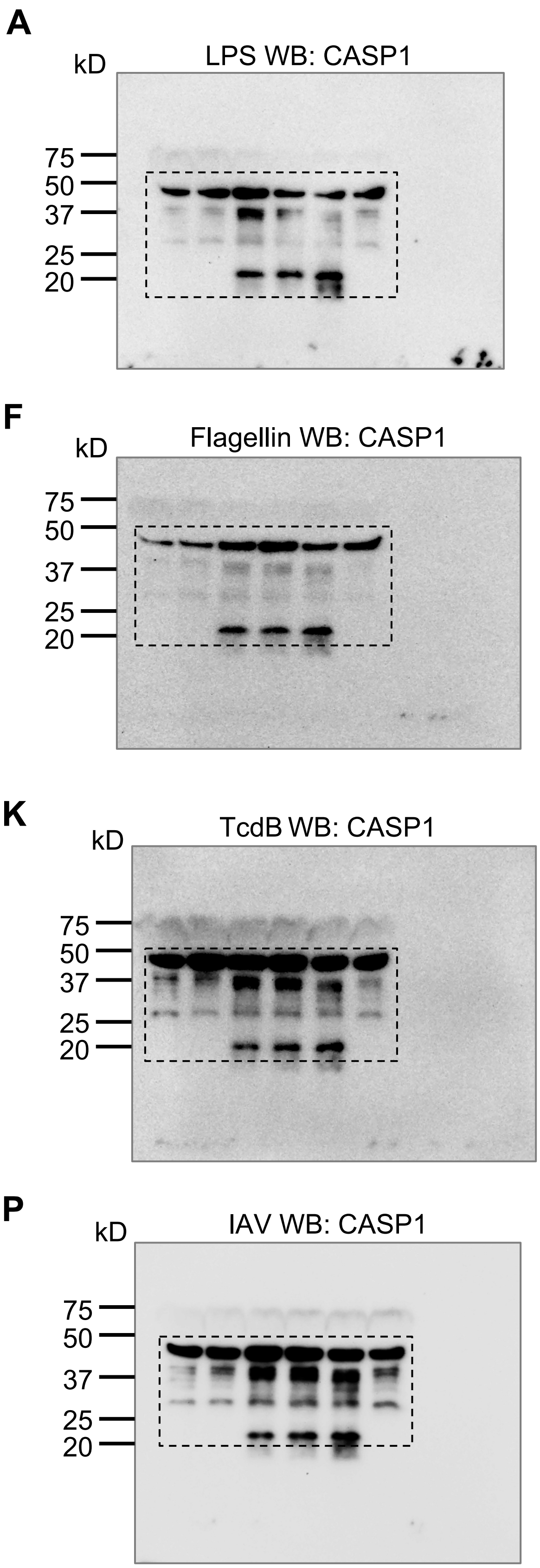

Supplementary Figure 2

A

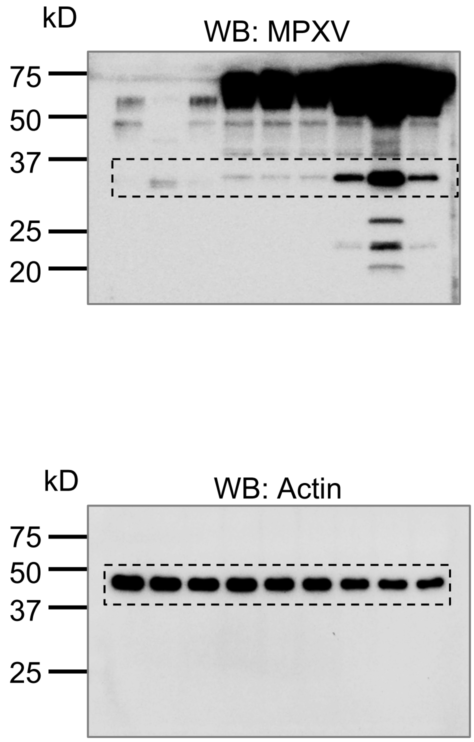

Supplementary Figure 4

C

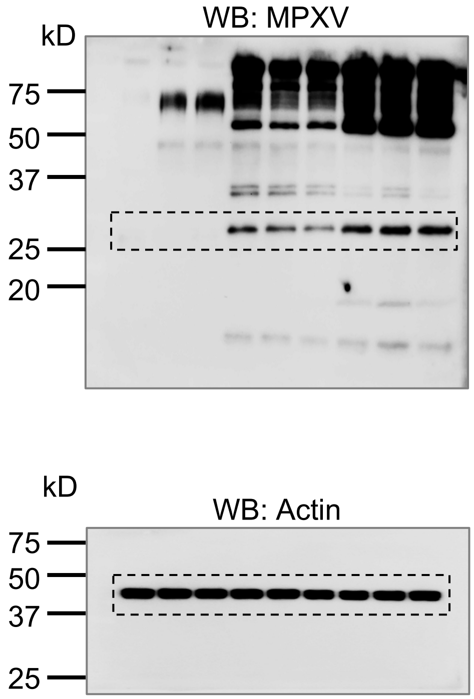

Supplementary Figure 5
