# Supplementary figures and images for: AIM2 drives inflammatory cell death and monkeypox pathogenesis
Source: Cell Mol Immunol. 2025 Nov 12;22(12):1615–28. doi: 10.1038/s41423-025-01367-7 (PMC12661009; doi:10.1038/s41423-025-01367-7)

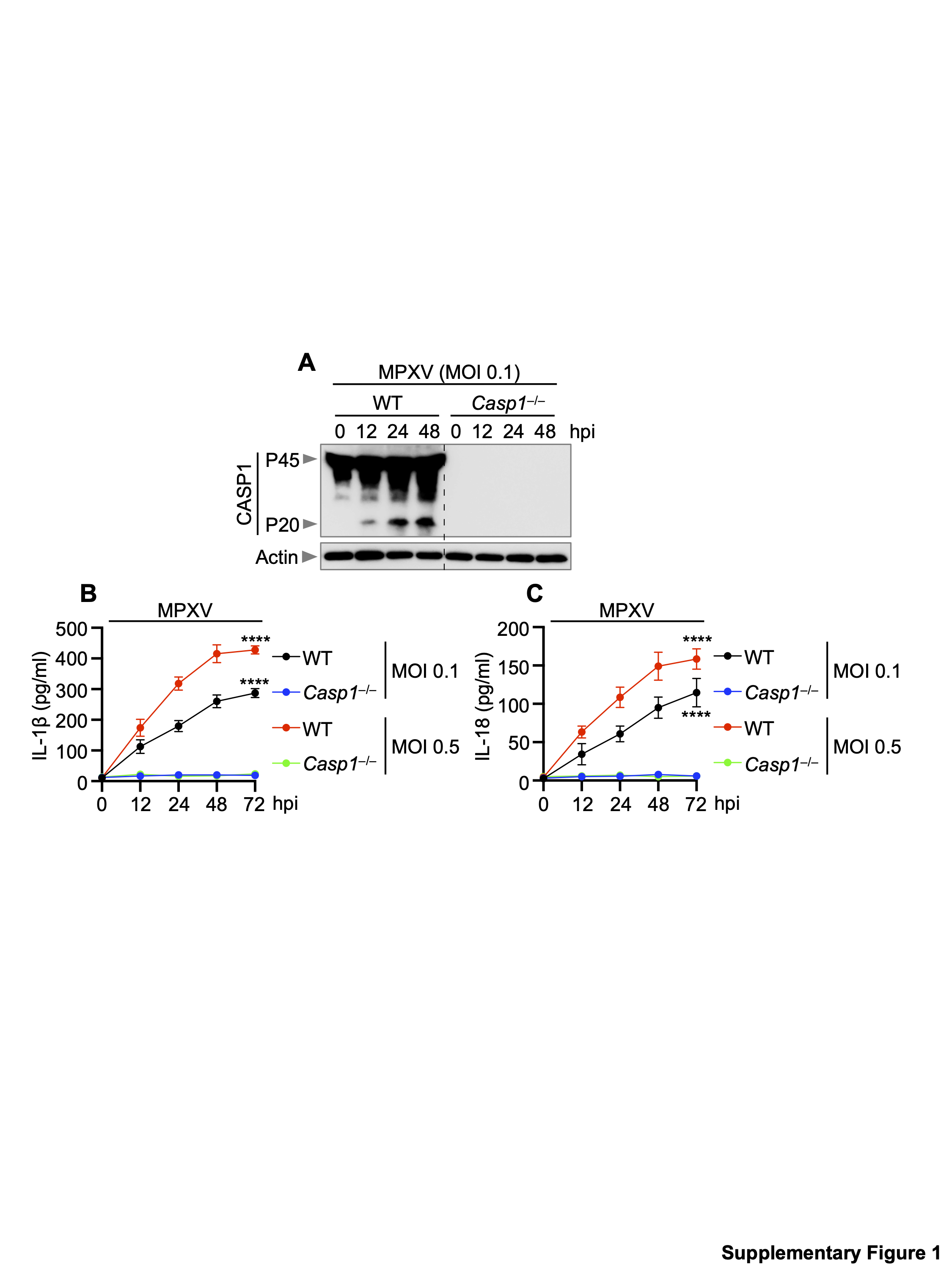

Supplement: Supplementary file 2 — Supplementary Figure 1 [file 41423_2025_1367_MOESM2_ESM.tif]

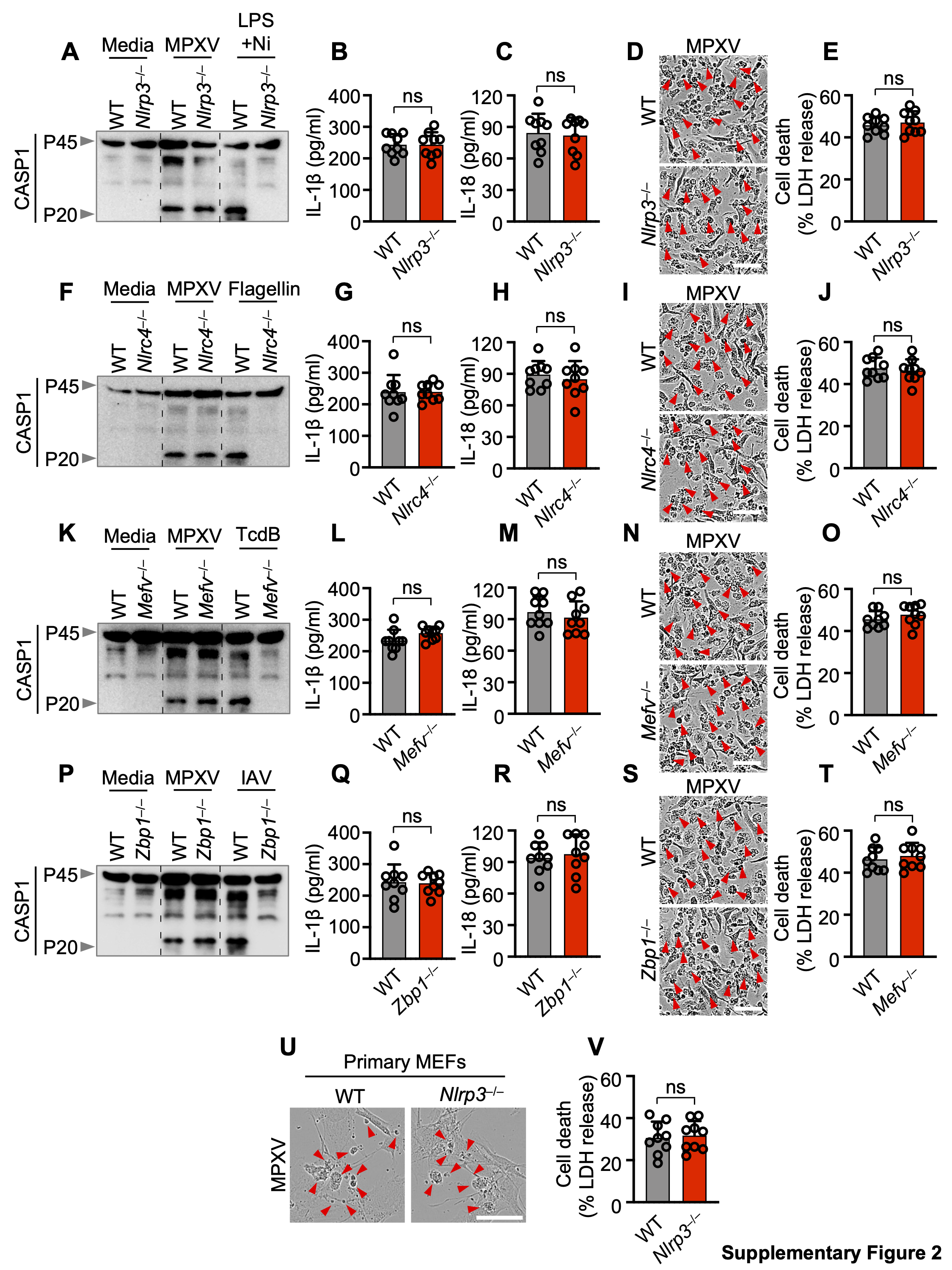

Supplement: Supplementary file 3 — Supplementary Figure 2 [file 41423_2025_1367_MOESM3_ESM.tif]

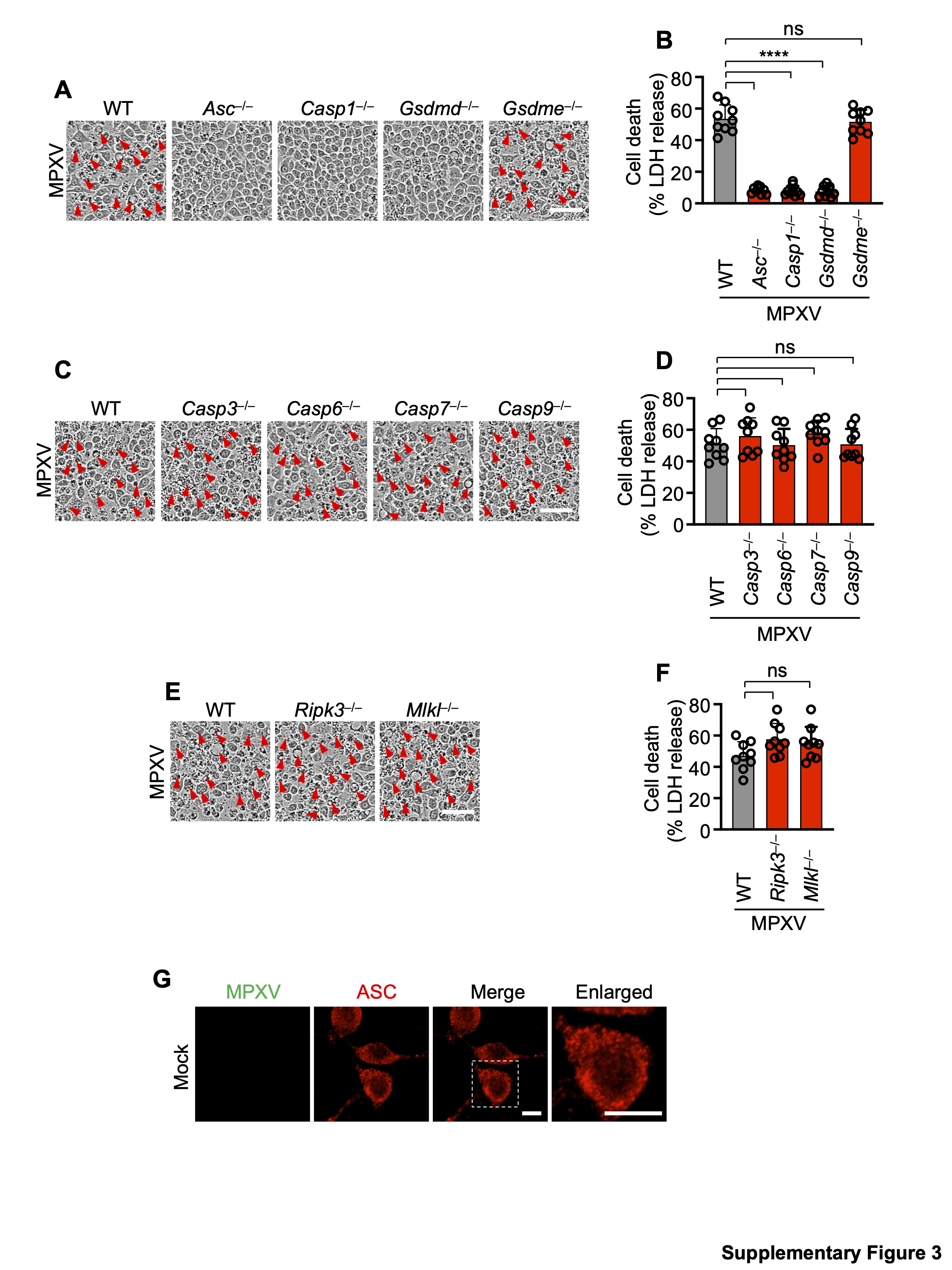

Supplement: Supplementary file 4 — Supplementary Figure 3 [file 41423_2025_1367_MOESM4_ESM.tif]

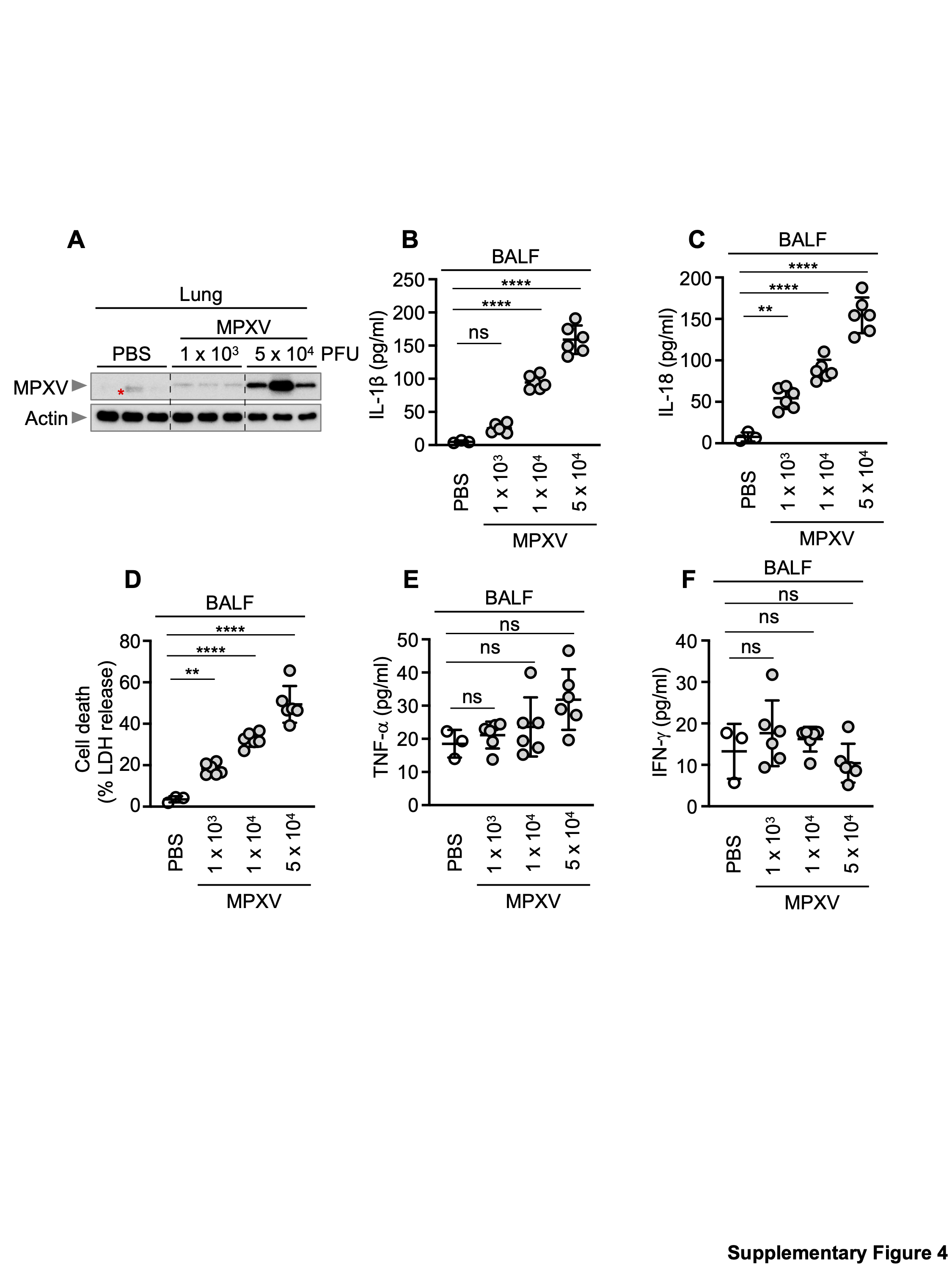

Supplement: Supplementary file 5 — Supplementary Figure 4 [file 41423_2025_1367_MOESM5_ESM.tif]

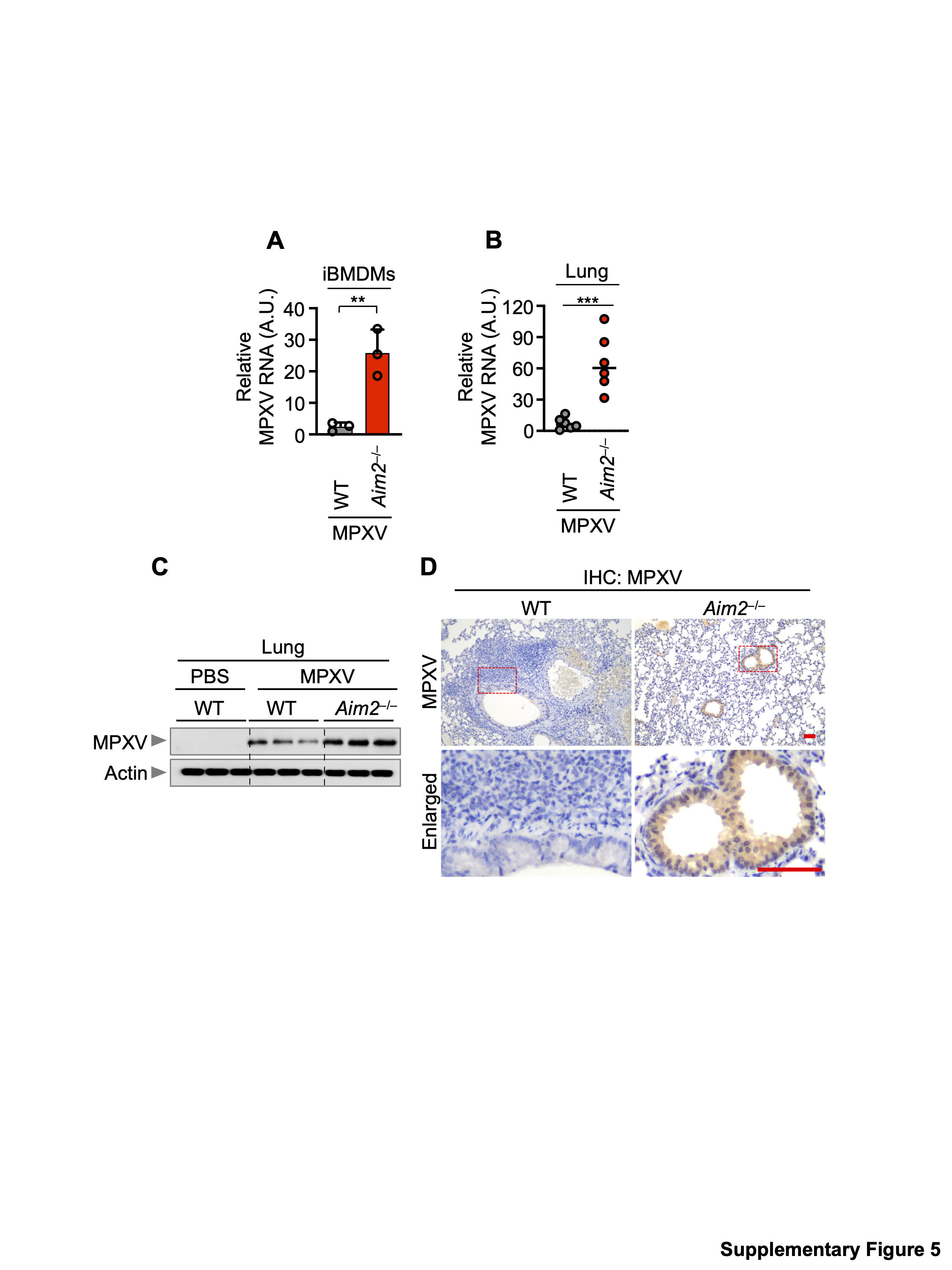

Supplement: Supplementary file 6 — Supplementary Figure 5 [file 41423_2025_1367_MOESM6_ESM.tif]
