# Supplementary material for: AIM2 drives inflammatory cell death and monkeypox pathogenesis
Source: Cell Mol Immunol. 2025 Nov 12;22(12):1615–28. doi: 10.1038/s41423-025-01367-7 (PMC12661009; doi:10.1038/s41423-025-01367-7)
Supplement: Supplementary file 7 — Supplementary Figure Legends [file 41423_2025_1367_MOESM7_ESM.docx]

**Supplementary Figure Legends**

**Figure S1. Induction of caspase-1 activation and cytokine release by monkeypox virus infection.**

(**A**) Immunoblot analysis of procaspase-1 (CASP1; P45) and cleaved CASP1 (P20) in wild-type (WT) or *Casp1*^−/−^ bone marrow-derived macrophages (BMDMs) after infection with monkeypox virus (MPXV). Time is denoted as h post infection (hpi). The data provided are representative of at least three independent experiments. (**B, C**) IL-1β (**B**) and IL-18 (**C**) release assessment in WT or *Casp1*^−/−^ BMDMs over a specified time course following MPXV infection. The data presented are expressed as the means ± s.e.m.s from three independent experiments. *****P* < 0.0001 (two-way ANOVA with Tukey’s multiple comparison test).

**Figure S2. Monkeypox virus infections do not activate the NLRP3, NLRC4, Pyrin, or ZBP1 inflammasome.**

(**A–E**) Immunoblot analysis of CASP1 (**A**), IL-1β release (**B**), IL-18 release (**C**), cell death images (**D**), and LDH release (**E**) from WT or *Nlrp3*^−/−^ bone marrow-derived macrophages (BMDMs) after monkeypox virus (MPXV) infection (MOI of 0.1 for 24 h) or lipopolysaccharide plus nigericin (LPS + Ni) treatment. (**F–J**) Immunoblot analysis of CASP1 (**F**), IL-1β release (**G**), IL-18 release (**H**), cell death (**I**), and LDH release (**J**) from WT or *Nlrc4*^−/−^ BMDMs after MPXV (MOI of 0.1 for 24 h) or flagellin transfection. (**K–O**) Immunoblot analysis of CASP1 (**K**), IL-1β release (**L**), IL-18 release (**M**), cell death images (**N**), and LDH release (**O**) from WT or *Mefv*^−/−^ BMDMs after MPXV infection (MOI of 0.1 for 24 h) or TcdB transfection. (**P–T**) Immunoblot analysis of CASP1 (**P**), IL-1β release (**Q**), IL-18 release (**R**), cell death images (**S**), and LDH release (**T**) from WT or *Zbp1*^−/−^ BMDMs after MPXV (MOI of 0.1 for 24 h) or IAV infection. (**U, V**) Representative images of cell death (**U**) and LDH release (**V**) in WT and *Nlrp3*⁻^/^⁻ mouse ear fibroblasts (MEFs) following MPXV infection (MOI 0.1, 24 h). (A), (F), (K), and (P) represent data from at least three independent experiments. (B), (C), (E), (G), (H), (J), (L), (M), (O), (Q), (R), (T), (V) Data are presented as the means ± s.e.m.s “ns,” not significant (two-tailed *t* test; *n* = 9 from 3 biologically independent samples). (D), (I), (N), (S), and (U) show images representative of a minimum of three independent experiments. Scale bar: 50 μm.

**Figure S3. ASC, caspase-1, and GSDMD are key components of AIM2-mediated inflammatory programmed cell death in response to monkeypox virus infection.**

(**A, B**) Evaluation of cell death (**A**) and lactate dehydrogenase (LDH) release (**B**) in wild-type (WT), *Asc*^−/−^, *Casp1*^−/−^, *Gsdmd*^−/−^, or *Gsdme*^−/−^ immortalized bone marrow-derived macrophages (iBMDMs) following monkeypox virus (MPXV) infection. (**C, D**) Evaluation of cell death (**C**) and LDH release (**D**) in WT, *Casp3*^−/−^, *Casp6*^−/−^, *Casp7*^−/−^, or *Casp9*^−/−^ iBMDMs following MPXV infection. (**E, F**) Evaluation of cell death (**E**) and LDH release (**F**) in WT, *Ripk3*^−/−^, or *Mlkl*^−/−^ iBMDMs following MPXV infection. (**G**) Immunofluorescence images of uninfected WT BMDMs showing ASC and MPXV staining. Scale bars, 5 μm. (A), (C), (E), and (G) show images representative of a minimum of three independent experiments. Scale bar: 50 μm. (B), (D), (F) Data are presented as the means ± s.e.m.s “ns,” not significant; *****P* < 0.0001 (one-way ANOVA with Dunnett’s multiple comparisons test; *n* = 9 from 3 biologically independent samples).

**Figure S4. Induction of proinflammatory cytokines by monkeypox virus infection in C57BL/6J mice.**

(**A**) Immunoblot analysis of monkeypox virus (MPXV) in lung samples from 8-week-old C57BL/6J wild-type (WT) mice 10 days after MPXV infection. Each lane represents an independent biological replicate. The red asterisk indicates a nonspecific band. (**B–F**) Levels of IL-1β (**B**), IL-18 (**C**), LDH (**D**), TNF-β (**E**), and IFN-γ (**F**) released into the bronchoalveolar lavage fluid (BALF) of WT mice on day 10 after MPXV infection (10^3^, 10^4^, or 5 × 10^4^ PFU). Each symbol corresponds to an individual mouse. The data were combined from two independent experiments and are presented as the means ± s.e.m.s “ns,” not significant. ***P* < 0.01, ****P* < 0.001, and *****P* < 0.0001 are the results of one-way ANOVA with Dunnett’s multiple comparisons test.

**Figure S5. AIM2 restricts MPXV replication and dissemination *in vitro* and *in vivo*.**

(**A**) Quantitative PCR analysis of the MPXV *F3L* gene in wild-type (WT) and *Aim2*⁻^/^⁻ immortalized bone marrow–derived macrophages (iBMDMs) at 24 h post infection (MOI = 0.1). Viral genome copy numbers were normalized to host *β-actin*. The data are presented as the means ± s.e.m.s; ***P* < 0.01 (two-tailed *t* test; *n* = 3 biologically independent samples). (**B**) Quantitative PCR analysis of *F3L* gene expression in lung tissues collected from WT and *Aim2*⁻^/^⁻ mice on day 5 post infection. Each symbol represents an individual animal. Data are combined from two independent experiments and expressed as the mean ± s.e.m. ****P* < 0.01 (two-tailed *t* test). (**C**) Immunoblot analysis of MPXV proteins in lung homogenates from infected WT and *Aim2*⁻^/^⁻ mice. Each lane represents an individual biological replicate. (**D**) Immunohistochemical (IHC) staining of MPXV antigens in lung sections from WT and *Aim2*⁻^/^⁻ mice on day 5 postinfection. Images are representative of five independent animals per group. Scale bars, 0.1 mm.
